# Supplementary material for: The human origin recognition complex is essential for pre-RC assembly, mitosis, and maintenance of nuclear structure
Source: eLife. 2021 Feb 1;10:e61797. doi: 10.7554/eLife.61797 (PMC7877914; doi:10.7554/eLife.61797)
Supplement: Figure 1—source data 1. — Log fold depletion (LFC) for ORC1 tiling-sgRNA CRISPR screen by MAGeCK HCT116 replicate 1. [file elife-61797-fig1-data1.docx]

Figure 1- source data 1

**Figure 1a - Log fold depletion (LFC) for ORC1 tiling-sgRNA CRISPR screen by MAGeCK
HCT116 replicate 1**

| aa Position | LFC |
| --- | --- |
| 6 | 0.33092 |
| 7 | 0.69533 |
| 7 | 0.26492 |
| 12 | 0.68095 |
| 13 | 0.91346 |
| 13 | 0.8535 |
| 14 | 1.1759 |
| 16 | 0.92325 |
| 19 | 1.1967 |
| 22 | 0.85531 |
| 22 | 0.48377 |
| 36 | 0.44984 |
| 44 | 1.3116 |
| 45 | 1.2655 |
| 46 | 1.1019 |
| 49 | 1.2545 |
| 53 | 1.0312 |
| 54 | 1.0564 |
| 54 | 0.9325 |
| 64 | 1.2523 |
| 65 | -0.038691 |
| 80 | 0.91989 |
| 81 | 0.41418 |
| 82 | 1.837 |
| 85 | 2.149 |
| 92 | 0.86371 |
| 97 | 1.5514 |
| 97 | 0.46015 |
| 98 | 0.82775 |
| 99 | 2.2625 |
| 100 | 0.89605 |
| 100 | 0.38922 |
| 102 | 1.1136 |
| 104 | 0.67353 |
| 106 | 1.9585 |
| 107 | 1.3486 |
| 109 | 0.13117 |
| 111 | 1.305 |
| 115 | 1.4165 |
| 119 | 0.42772 |
| 120 | 0.8317 |
| aa Position | LFC |
| 121 | 0.0052628 |
| 123 | 1.7461 |
| 127 | 0.99934 |
| 131 | 1.2181 |
| 139 | 0.92417 |
| 140 | 0.1502 |
| 141 | 1.3257 |
| 141 | 1.2024 |
| 142 | 1.3575 |
| 147 | 0.48919 |
| 157 | 0.59756 |
| 161 | 0.58416 |
| 163 | 0.84462 |
| 169 | 1.1937 |
| 171 | 1.3167 |
| 171 | 0.79317 |
| 181 | 0.55504 |
| 186 | 1.0479 |
| 188 | 1.0715 |
| 191 | 1.1881 |
| 191 | 0.5431 |
| 194 | 0.16822 |
| 199 | 0.99015 |
| 201 | 1.6643 |
| 202 | 1.2756 |
| 205 | 0.94211 |
| 205 | 0.80827 |
| 205 | 0.47514 |
| 206 | 0.53045 |
| 208 | 0.63963 |
| 211 | 0.29823 |
| 212 | 0.76959 |
| 219 | 0.83692 |
| 220 | 0.85777 |
| 221 | 0.23417 |
| 224 | 1.5961 |
| 227 | 1.504 |
| 228 | 1.2594 |
| 228 | 0.51705 |
| 230 | 0.75034 |
| 232 | 0.89187 |
| aa Position | LFC |
| 232 | 0.79597 |
| 233 | 0.81863 |
| 233 | 0.15423 |
| 234 | -0.25508 |
| 235 | 0.35987 |
| 236 | 0.74539 |
| 246 | 0.99435 |
| 247 | 1.0346 |
| 250 | 0.56239 |
| 250 | 0.72653 |
| 253 | 0.97773 |
| 256 | 1.3158 |
| 256 | 1.0432 |
| 257 | 1.4672 |
| 261 | 0.84981 |
| 261 | 0.63173 |
| 263 | 0.44911 |
| 266 | 0.36486 |
| 269 | 0.88346 |
| 274 | 1.3212 |
| 276 | 0.81059 |
| 282 | 0.97734 |
| 287 | 2.0313 |
| 290 | 0.77826 |
| 294 | 0.87152 |
| 294 | 0.73395 |
| 295 | 0.70767 |
| 296 | 1.1905 |
| 298 | 1.4142 |
| 302 | 1.4366 |
| 306 | 1.2511 |
| 314 | 0.95873 |
| 319 | 0.857 |
| 322 | 1.8305 |
| 322 | 0.63548 |
| 329 | 0.86636 |
| 329 | 0.8333 |
| 337 | 0.69137 |
| 338 | 1.224 |
| 338 | 0.97424 |
| 338 | 1.1188 |
| 339 | 1.2514 |
| 339 | 0.64573 |
| 340 | 1.2668 |
| 344 | 0.96786 |
| 351 | 0.43982 |
| 352 | 0.83254 |
| 370 | 1.0075 |
| 370 | 0.71164 |
| 375 | 0.77047 |
| 378 | 0.89288 |
| 378 | 0.84098 |
| 378 | -0.0073084 |
| 381 | 1.0547 |
| 387 | 0.70679 |
| 389 | 1.003 |
| 406 | 0.44673 |
| 414 | 0.65936 |
| 422 | 0.50767 |
| 429 | 1.1122 |
| 430 | 1.3534 |
| 431 | 0.83864 |
| 432 | 1.3448 |
| 432 | 1.0793 |
| 432 | 1.0234 |
| 434 | 0.85065 |
| 438 | 1.1843 |
| 438 | 0.84504 |
| 438 | 0.65924 |
| 442 | 1.3032 |
| 444 | 0.82965 |
| 448 | 1.0035 |
| 452 | 0.83466 |
| 453 | 1.6585 |
| 455 | 1.309 |
| 455 | 0.67359 |
| 466 | 1.7294 |
| 469 | 1.4908 |
| 472 | 1.2042 |
| 474 | 1.1633 |
| 476 | 1.1314 |
| 476 | 0.53462 |
| 479 | 1.3896 |
| 480 | 0.59103 |
| 483 | 0.69163 |
| 483 | 1.6794 |
| 485 | 0.35024 |
| 486 | 0.95462 |
| 486 | 0.65669 |
| 487 | 1.1802 |
| 503 | 1.4224 |
| 504 | 1.3275 |
| 504 | 1.305 |
| 506 | -0.037122 |
| 507 | 1.2857 |
| 507 | 0.85349 |
| 513 | 0.097611 |
| 515 | 0.14855 |
| 523 | 1.2369 |
| 524 | 1.5599 |
| 533 | 0.97171 |
| 534 | 2.5055 |
| 535 | 1.0706 |
| 535 | 0.70686 |
| 536 | 1.3958 |
| 537 | 2.1801 |
| 538 | 1.1566 |
| 544 | 2.0898 |
| 550 | 1.7664 |
| 552 | 0.99376 |
| 557 | 1.2885 |
| 557 | 0.92842 |
| 559 | 0.87543 |
| 563 | 1.2977 |
| 564 | 1.1032 |
| 564 | 1.322 |
| 564 | 0.74505 |
| 566 | 1.1243 |
| 571 | 1.7316 |
| 578 | 1.652 |
| 578 | 0.78306 |
| 578 | 0.68717 |
| 579 | 1.0189 |
| 591 | 0.47963 |
| 594 | 1.4336 |
| 596 | 1.886 |
| 597 | 0.62923 |
| 598 | 0.43504 |
| 605 | 0.9179 |
| 606 | 0.92153 |
| 609 | 2.0972 |
| 609 | 1.3994 |
| 609 | 1.5667 |
| 613 | 1.8274 |
| 616 | 1.1622 |
| 616 | 0.57912 |
| 625 | 1.543 |
| 636 | 0.32483 |
| 640 | 0.80383 |
| 641 | 1.4182 |
| 642 | 1.1091 |
| 642 | 0.91642 |
| 643 | 0.82773 |
| 645 | 1.2811 |
| 647 | 2.028 |
| 647 | 0.80994 |
| 647 | 0.10588 |
| 651 | 1.0811 |
| 653 | 1.8756 |
| 655 | 1.621 |
| 659 | 0.5843 |
| 661 | 0.94834 |
| 663 | 1.5945 |
| 663 | 0.89777 |
| 676 | 2.596 |
| 680 | 1.544 |
| 682 | 1.8969 |
| 682 | 1.4024 |
| 686 | 0.75272 |
| 688 | 1.9089 |
| 690 | 1.6065 |
| 691 | 1.4428 |
| 694 | 0.9109 |
| 694 | 0.55583 |
| 695 | 1.2068 |
| 701 | 0.84865 |
| 704 | 1.4796 |
| 706 | 1.9331 |
| 707 | 0.50215 |
| 718 | 1.2985 |
| 720 | 0.27889 |
| 724 | 1.7701 |
| 724 | 1.0477 |
| 731 | 1.4015 |
| 738 | 2.1101 |
| 738 | 0.5302 |
| 740 | 0.83631 |
| 742 | 0.95713 |
| 742 | 0.32664 |
| 744 | 1.8941 |
| 744 | 0.6337 |
| 745 | 0.56273 |
| 746 | 0.96183 |
| 749 | 1.8197 |
| 749 | 1.0282 |
| 751 | 1.2491 |
| 751 | 0.8297 |
| 752 | 0.64249 |
| 762 | 1.6313 |
| 772 | 0.92232 |
| 779 | 1.4635 |
| 782 | 0.9437 |
| 783 | 1.3977 |
| 785 | 1.4255 |
| 787 | 1.0493 |
| 787 | 1.4816 |
| 788 | 1.4604 |
| 805 | 2.1629 |
| 806 | 1.8209 |
| 806 | 1.4984 |
| 814 | 0.62351 |
| 815 | 0.48612 |
| 816 | 1.5754 |
| 816 | 1.2591 |
| 817 | 1.8799 |
| 817 | 1.3391 |
| 821 | 2.1289 |
| 823 | 2.4139 |
| 823 | 1.6237 |
| 823 | 0.69328 |
| 827 | 2.3117 |
| 830 | 2.0211 |
| 831 | 1.3977 |
| 832 | 1.723 |
| 833 | 1.9529 |
| 835 | 0.8051 |
| 838 | 0.79162 |
| 838 | 0.39063 |
| 841 | 1.7497 |
| 841 | 1.1677 |
| 843 | 1.6337 |
| 844 | 1.2783 |
| 848 | 0.50594 |
| 852 | 1.2148 |
