# Supplementary material for: The human origin recognition complex is essential for pre-RC assembly, mitosis, and maintenance of nuclear structure
Source: eLife. 2021 Feb 1;10:e61797. doi: 10.7554/eLife.61797 (PMC7877914; doi:10.7554/eLife.61797)
Supplement: Figure 1—source data 2. — Log fold depletion (LFC) for ORC1 tiling-sgRNA CRISPR screen by MAGeCK RPE-1. [file elife-61797-fig1-data2.docx]

Figure 1 – source data 2

**Figure 1b - Log fold depletion (LFC) for ORC1 tiling-sgRNA CRISPR screen by MAGeCK
RPE-1**

| aa Position | LFC |
| --- | --- |
| 6 | 3.4169 |
| 7 | 4.2387 |
| 7 | 1.7923 |
| 12 | 1.7836 |
| 13 | 4.6821 |
| 13 | 5.7689 |
| 14 | 5.1053 |
| 16 | 6.1859 |
| 19 | 7.8669 |
| 22 | 5.9063 |
| 22 | 3.612 |
| 36 | 5.217 |
| 44 | 8.768 |
| 45 | 6.5035 |
| 46 | 6.1747 |
| 49 | 5.339 |
| 53 | 7.2304 |
| 54 | 7.5918 |
| 54 | 8.2188 |
| 64 | 8.0795 |
| 65 | 1.8077 |
| 80 | 3.1 |
| 81 | 9.269 |
| 82 | 7.1963 |
| 85 | 8.0847 |
| 92 | 5.0809 |
| 97 | 6.2786 |
| 97 | 5.4532 |
| 98 | 5.9344 |
| 99 | 7.1089 |
| 100 | 12.162 |
| 100 | 6.1766 |
| 102 | 12.029 |
| 104 | 1.1235 |
| 106 | 12.614 |
| 107 | 8.2485 |
| 109 | 3.7777 |
| 111 | 11.711 |
| 115 | 3.5854 |
| 119 | 5.5331 |
| 120 | 6.4649 |
| aa Position | LFC |
| 121 | 3.3942 |
| 123 | 12.909 |
| 127 | 7.2533 |
| 131 | 7.4318 |
| 139 | 3.82 |
| 140 | 5.7012 |
| 141 | 5.0996 |
| 141 | 3.9769 |
| 142 | 7.8788 |
| 147 | 1.3299 |
| 157 | 7.2406 |
| 161 | 5.7556 |
| 163 | 7.2526 |
| 169 | 10.102 |
| 171 | 8.6239 |
| 171 | 6.5605 |
| 181 | 5.3115 |
| 186 | 6.2616 |
| 188 | 4.7075 |
| 191 | 4.7579 |
| 191 | 4.8758 |
| 194 | 4.0575 |
| 199 | 3.4937 |
| 201 | 4.5889 |
| 202 | 3.9896 |
| 205 | 5.4325 |
| 205 | 4.607 |
| 205 | 2.6137 |
| 206 | 2.5073 |
| 208 | 5.0423 |
| 211 | 3.7739 |
| 212 | 3.3508 |
| 219 | 1.9165 |
| 220 | 2.7336 |
| 221 | 3.5933 |
| 224 | 5.2164 |
| 227 | 8.8373 |
| 228 | 4.5779 |
| 228 | 2.8499 |
| 230 | 2.5238 |
| 232 | 2.8764 |
| aa Position | LFC |
| 232 | 2.0584 |
| 233 | 2.8709 |
| 233 | 3.6099 |
| 234 | 3.3587 |
| 235 | 2.6409 |
| 236 | 3.0552 |
| 246 | 4.8626 |
| 247 | 4.4822 |
| 250 | 5.6173 |
| 250 | 2.8986 |
| 253 | 3.5546 |
| 256 | 4.937 |
| 256 | 3.0389 |
| 257 | 6.108 |
| 261 | 6.2369 |
| 261 | 1.6867 |
| 263 | 4.1489 |
| 266 | 3.3618 |
| 269 | 4.9344 |
| 274 | 5.0879 |
| 276 | 3.337 |
| 282 | 5.5573 |
| 287 | 2.7659 |
| 290 | 5.7192 |
| 294 | 4.4093 |
| 294 | 2.7604 |
| 295 | 5.2647 |
| 296 | 5.3046 |
| 298 | 2.6183 |
| 302 | 4.5013 |
| 306 | 4.6514 |
| 314 | 5.028 |
| 319 | 4.8043 |
| 322 | 6.6502 |
| 322 | 6.9819 |
| 329 | 5.651 |
| 329 | 4.67 |
| 337 | 3.5204 |
| 338 | 6.0732 |
| 338 | 3.3352 |
| 338 | 4.0965 |
| 339 | 4.2013 |
| 339 | 4.4502 |
| 340 | 7.1303 |
| 344 | 5.4783 |
| 351 | 2.3272 |
| 352 | 4.0571 |
| 370 | 3.1827 |
| 370 | 2.725 |
| 375 | 5.1738 |
| 378 | 4.113 |
| 378 | 3.2491 |
| 378 | 3.0439 |
| 381 | 3.9087 |
| 387 | 3.8362 |
| 389 | 2.3983 |
| 406 | 3.2737 |
| 414 | 5.1497 |
| 422 | 4.0097 |
| 429 | 10.326 |
| 430 | 7.1611 |
| 431 | 4.3631 |
| 432 | 5.1022 |
| 432 | 4.6887 |
| 432 | 5.477 |
| 434 | 2.6484 |
| 438 | 6.3022 |
| 438 | 5.3974 |
| 438 | 3.7384 |
| 442 | 8.0878 |
| 444 | 8.237 |
| 448 | 3.5756 |
| 452 | 5.8678 |
| 453 | 6.0531 |
| 455 | 6.5653 |
| 455 | 3.8807 |
| 466 | 3.2696 |
| 469 | 3.626 |
| 472 | 4.0131 |
| 474 | 2.1424 |
| 476 | 5.4949 |
| 476 | 4.4693 |
| 479 | 4.9515 |
| 480 | 3.3626 |
| 483 | 1.9604 |
| 483 | 6.7523 |
| 485 | 4.7052 |
| 486 | 4.4019 |
| 486 | 4.9831 |
| 487 | 4.9309 |
| 503 | 11.701 |
| 504 | 8.4324 |
| 504 | 5.0103 |
| 506 | 7.1541 |
| 507 | 7.9085 |
| 507 | 7.5084 |
| 513 | 3.6669 |
| 515 | 1.291 |
| 523 | 12.896 |
| 524 | 6.309 |
| 533 | 4.6444 |
| 534 | 8.9241 |
| 535 | 6.3613 |
| 535 | 4.8524 |
| 536 | 7.7908 |
| 537 | 7.4299 |
| 538 | 12.449 |
| 544 | 8.6844 |
| 550 | 8.2834 |
| 552 | 3.4378 |
| 557 | 7.1188 |
| 557 | 2.6641 |
| 559 | 3.155 |
| 563 | 7.1033 |
| 564 | 6.9284 |
| 564 | 9.6853 |
| 564 | 7.4853 |
| 566 | 6.1511 |
| 571 | 7.1457 |
| 578 | 5.0121 |
| 578 | 6.3388 |
| 578 | 5.9325 |
| 579 | 10.039 |
| 591 | 1.0176 |
| 594 | 8.7497 |
| 596 | 6.8926 |
| 597 | 8.9463 |
| 598 | 4.9899 |
| 605 | 3.3109 |
| 606 | 5.6766 |
| 609 | 6.6417 |
| 609 | 7.5446 |
| 609 | 5.7128 |
| 613 | 9.0618 |
| 616 | 7.9291 |
| 616 | 7.145 |
| 625 | 7.9398 |
| 636 | 9.817 |
| 640 | 7.412 |
| 641 | 6.916 |
| 642 | 7.355 |
| 642 | 7.7851 |
| 643 | 3.0814 |
| 645 | 2.5892 |
| 647 | 5.7849 |
| 647 | 4.0484 |
| 647 | 2.707 |
| 651 | 12.374 |
| 653 | 9.1488 |
| 655 | 8.721 |
| 659 | 6.1817 |
| 661 | 6.281 |
| 663 | 7.061 |
| 663 | 6.8521 |
| 676 | 11.904 |
| 680 | 12.222 |
| 682 | 12.451 |
| 682 | 8.8901 |
| 686 | 10.397 |
| 688 | 8.5218 |
| 690 | 4.6005 |
| 691 | 9.7152 |
| 694 | 3.1868 |
| 694 | 2.1072 |
| 695 | 4.1749 |
| 701 | 8.8247 |
| 704 | 10.115 |
| 706 | 12.792 |
| 707 | 2.0691 |
| 718 | 9.3596 |
| 720 | 2.3684 |
| 724 | 12.051 |
| 724 | 12.286 |
| 731 | 11.914 |
| 738 | 6.2214 |
| 738 | 3.0039 |
| 740 | 3.9746 |
| 742 | 3.697 |
| 742 | 1.9826 |
| 744 | 4.2938 |
| 744 | 5.6513 |
| 745 | 1.8207 |
| 746 | 7.495 |
| 749 | 5.0607 |
| 749 | 5.1202 |
| 751 | 7.9978 |
| 751 | 5.0735 |
| 752 | 4.6838 |
| 762 | 9.1 |
| 772 | 7.4647 |
| 779 | 7.5046 |
| 782 | 7.548 |
| 783 | 6.5335 |
| 785 | 6.3735 |
| 787 | 7.4881 |
| 787 | 8.6228 |
| 788 | 5.2636 |
| 805 | 6.7061 |
| 806 | 9.2186 |
| 806 | 10.888 |
| 814 | 2.5929 |
| 815 | 5.0309 |
| 816 | 6.6427 |
| 816 | 4.3301 |
| 817 | 5.5381 |
| 817 | 8.5464 |
| 821 | 12.589 |
| 823 | 7.3966 |
| 823 | 12.237 |
| 823 | 12.505 |
| 827 | 4.7223 |
| 830 | 6.999 |
| 831 | 8.3878 |
| 832 | 8.1486 |
| 833 | 5.1505 |
| 835 | 7.202 |
| 838 | 7.807 |
| 838 | 5.4647 |
| 841 | 6.0527 |
| 841 | 3.9506 |
| 843 | 6.9625 |
| 844 | 7.9138 |
| 848 | 4.9465 |
| 852 | 6.1279 |
