# Supplementary material for: The human origin recognition complex is essential for pre-RC assembly, mitosis, and maintenance of nuclear structure
Source: eLife. 2021 Feb 1;10:e61797. doi: 10.7554/eLife.61797 (PMC7877914; doi:10.7554/eLife.61797)
Supplement: Figure 2—source data 1. — Log fold depletion (LFC) for ORC2 tiling-sgRNA CRISPR screen by MAGeCK HCT116 replicate 1. [file elife-61797-fig2-data1.docx]

Figure 2-source data 1

**Figure 2a - Log fold depletion (LFC) for ORC2 tiling-sgRNA CRISPR screen by MAGeCK
HCT116 replicate 1**

| aa postion | LFC |
| --- | --- |
| 6 | 0.37482 |
| 9 | 1.5392 |
| 10 | 0.79536 |
| 14 | 1.0913 |
| 14 | 0.94534 |
| 27 | 0.65668 |
| 40 | 1.5134 |
| 47 | 0.58168 |
| 48 | 0.15377 |
| 48 | 0.87793 |
| 54 | 0.046535 |
| 55 | 1.0994 |
| 55 | 0.68492 |
| 59 | 0.65608 |
| 60 | 0.6455 |
| 63 | 1.2949 |
| 68 | 0.48792 |
| 71 | 0.96371 |
| 71 | 1.1229 |
| 85 | 0.6113 |
| 86 | 0.44439 |
| 87 | 0.30239 |
| 103 | 1.7749 |
| 119 | 0.95976 |
| 131 | 0.89985 |
| 137 | 0.43893 |
| 143 | 1.3909 |
| 165 | 0.82608 |
| 175 | 1.5569 |
| 186 | 1.4795 |
| 188 | 1.3628 |
| 189 | 0.89392 |
| 190 | 0.79003 |
| 190 | 2.2398 |
| 193 | 1.5095 |
| 197 | 0.90433 |
| 210 | 1.3882 |
| aa postion | LFC |
| 219 | 0.67337 |
| 223 | 1.4242 |
| 229 | 0.81239 |
| 256 | 1.4885 |
| 264 | 0.5128 |
| 275 | 0.99042 |
| 282 | 2.2368 |
| 282 | 1.2623 |
| 283 | 1.1571 |
| 284 | 1.1759 |
| 287 | 0.62488 |
| 298 | 1.5874 |
| 308 | 1.7298 |
| 311 | 0.7925 |
| 313 | 1.3551 |
| 313 | 1.4334 |
| 322 | 0.32027 |
| 330 | 1.1948 |
| 336 | 1.8559 |
| 337 | 0.99603 |
| 341 | 1.5395 |
| 353 | 0.81993 |
| 360 | 0.98505 |
| 360 | 1.3983 |
| 361 | 1.2309 |
| 367 | 1.8567 |
| 368 | 2.3377 |
| 372 | 1.0749 |
| 391 | 1.3106 |
| 392 | 1.0019 |
| 394 | 2.2697 |
| 398 | 1.0039 |
| 399 | 1.6556 |
| 406 | 1.6977 |
| 407 | 0.64639 |
| 421 | 1.7975 |
| 425 | 1.0392 |
| aa postion | LFC |
| 440 | 0.976 |
| 442 | 1.1442 |
| 455 | 1.8918 |
| 460 | 1.027 |
| 461 | 1.777 |
| 462 | 0.62127 |
| 466 | 1.4495 |
| 473 | 1.2993 |
| 473 | 2.1617 |
| 475 | 1.5853 |
| 478 | 2.5351 |
| 478 | 0.81746 |
| 497 | 1.2484 |
| 500 | 1.1771 |
| 500 | 0.93592 |
| 504 | 0.57932 |
| 512 | 0.34387 |
| 518 | 0.81763 |
| 518 | 1.9926 |
| 519 | 1.8655 |
| 519 | 1.992 |
| 526 | 3.3226 |
| 530 | 2.3476 |
| 530 | 1.488 |
| 534 | 0.10077 |
| 536 | 0.87374 |
| 536 | 0.88607 |
| 537 | 0.90199 |
| 537 | 0.55936 |
| 543 | 0.61454 |
| 560 | 0.8564 |
| 562 | 1.3986 |
| 567 | 0.64159 |
| 569 | 0.27278 |
| 572 | 0.52861 |
| 573 | 0.937 |
