# Supplementary material for: The human origin recognition complex is essential for pre-RC assembly, mitosis, and maintenance of nuclear structure
Source: eLife. 2021 Feb 1;10:e61797. doi: 10.7554/eLife.61797 (PMC7877914; doi:10.7554/eLife.61797)
Supplement: Figure 2—source data 2. — Log fold depletion (LFC) for ORC2 tiling-sgRNA CRISPR screen by MAGeCK RPE-1. [file elife-61797-fig2-data2.docx]

Figure 2 – source data 2

**Figure 2b - Log fold depletion (LFC) for ORC2 tiling-sgRNA CRISPR screen by MAGeCK
RPE-1**

| aa position | LFC |
| --- | --- |
| 6 | 4.6341 |
| 9 | 5.2695 |
| 10 | 4.6314 |
| 14 | 5.0894 |
| 14 | 6.1663 |
| 27 | 2.5443 |
| 40 | 4.4581 |
| 47 | 3.2748 |
| 48 | 4.89 |
| 48 | 2.9744 |
| 54 | 4.3041 |
| 55 | 5.6461 |
| 55 | 6.0719 |
| 59 | 3.7894 |
| 60 | 6.034 |
| 63 | 3.1586 |
| 68 | 4.5329 |
| 71 | 6.0924 |
| 71 | 3.8282 |
| 85 | 3.1777 |
| 86 | 5.0407 |
| 87 | 5.3197 |
| 103 | 6.1506 |
| 119 | 2.6374 |
| 131 | 6.8916 |
| 137 | 7.8893 |
| 143 | 6.2861 |
| 165 | 2.4198 |
| 175 | 8.1963 |
| 186 | 7.7187 |
| 188 | 8.2818 |
| 189 | 4.2633 |
| 190 | 7.5574 |
| 190 | 6.7924 |
| 193 | 6.1627 |
| 197 | 5.625 |
| 210 | 4.5255 |
| 219 | 3.5292 |
| 223 | 4.1474 |
| 229 | 5.2869 |
| aa position | LFC |
| 256 | 3.9687 |
| 264 | 6.2557 |
| 275 | 4.7094 |
| 282 | 6.8116 |
| 282 | 8.3464 |
| 283 | 7.1342 |
| 284 | 3.354 |
| 287 | 8.7863 |
| 298 | 4.1826 |
| 308 | 8.9999 |
| 311 | 7.923 |
| 313 | 7.4299 |
| 313 | 7.5656 |
| 322 | 7.8731 |
| 330 | 8.0922 |
| 336 | 9.0312 |
| 337 | 9.7921 |
| 341 | 10.006 |
| 353 | 8.6845 |
| 360 | 5.8013 |
| 360 | 2.1792 |
| 361 | 7.7683 |
| 367 | 9.3786 |
| 368 | 12.233 |
| 372 | 9.6479 |
| 391 | 9.2643 |
| 392 | 10.889 |
| 394 | 8.628 |
| 398 | 7.7843 |
| 399 | 1.8146 |
| 406 | 8.2641 |
| 407 | 5.7996 |
| 421 | 10.456 |
| 425 | 5.6052 |
| 440 | 5.3403 |
| 442 | 4.5241 |
| 455 | 5.3181 |
| 460 | 13.273 |
| 461 | 10.709 |
| 462 | 2.2824 |
| aa position | LFC |
| 466 | 5.745 |
| 473 | 4.88 |
| 473 | 5.2726 |
| 475 | 6.6707 |
| 478 | 8.1809 |
| 478 | 6.8894 |
| 497 | 11.987 |
| 500 | 8.7366 |
| 500 | 10.073 |
| 504 | 2.1998 |
| 512 | 7.5928 |
| 518 | 10.714 |
| 518 | 8.7859 |
| 519 | 6.4258 |
| 519 | 8.1018 |
| 526 | 8.468 |
| 530 | 10.041 |
| 530 | 7.2097 |
| 534 | 2.2741 |
| 536 | 8.3508 |
| 536 | 7.1845 |
| 537 | 7.772 |
| 537 | 6.8239 |
| 543 | 7.908 |
| 560 | 2.2276 |
| 562 | 5.7357 |
| 567 | 7.1598 |
| 569 | 3.377 |
| 572 | 3.8159 |
| 573 | 6.5287 |
