# Supplementary figures and images for: The human origin recognition complex is essential for pre-RC assembly, mitosis, and maintenance of nuclear structure
Source: eLife. 2021 Feb 1;10:e61797. doi: 10.7554/eLife.61797 (PMC7877914; doi:10.7554/eLife.61797)

## Slide 1
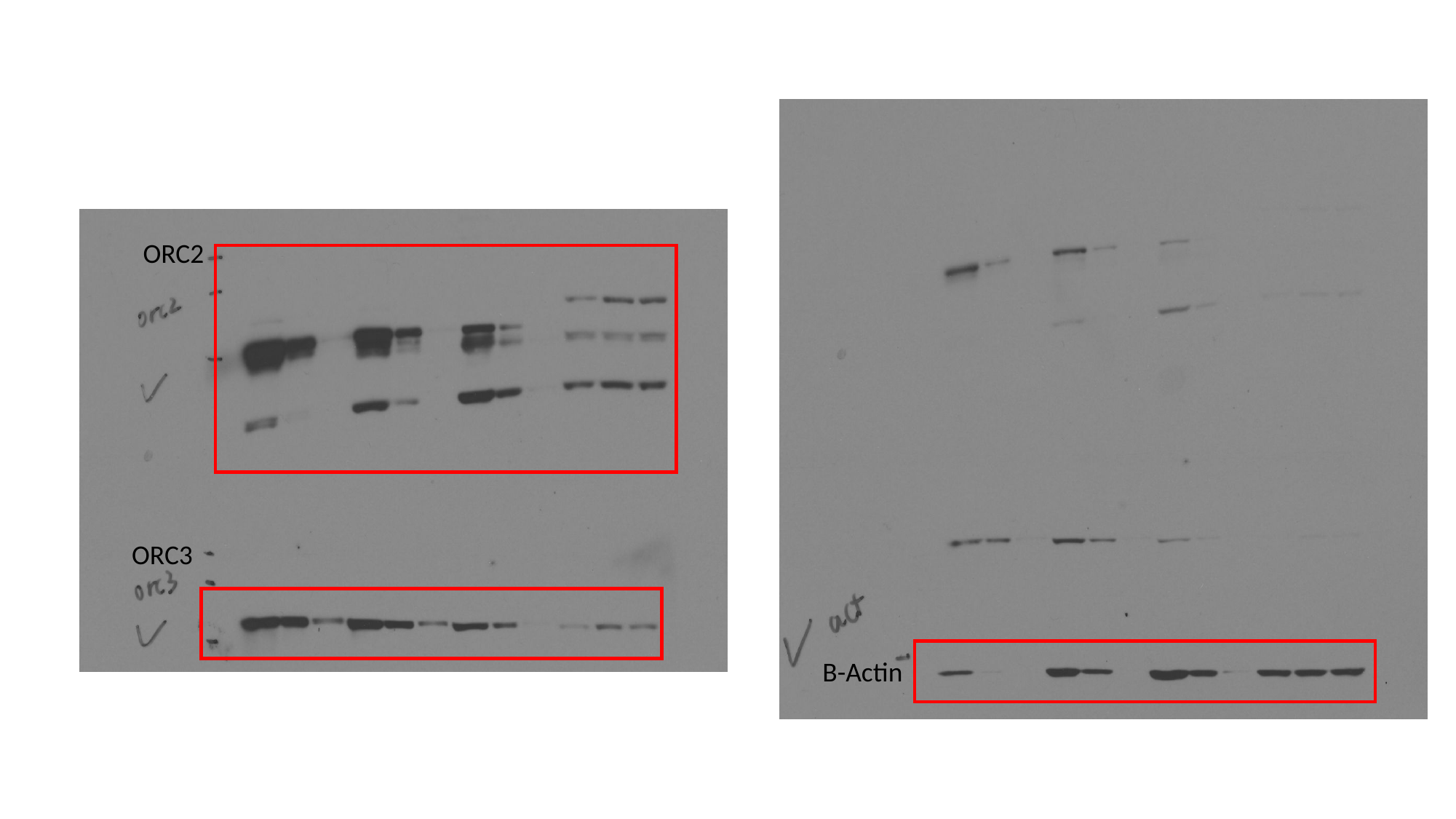

ORC2
ORC3
Β-Actin

Supplement: Figure 3—source data 1. [file elife-61797-fig3-data1.pptx]

## Slide 1
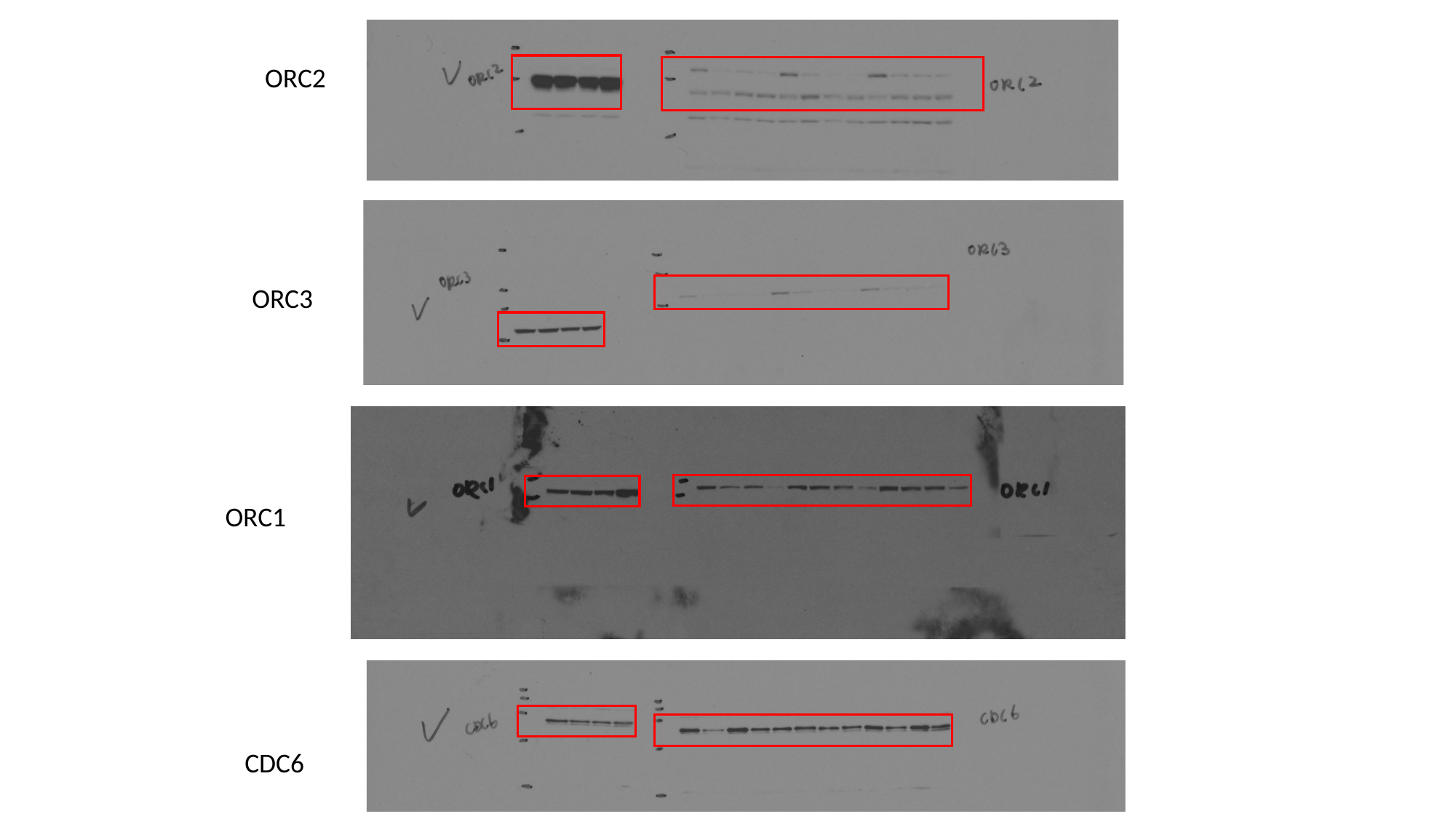

ORC2
ORC3
ORC1
CDC6

## Slide 2
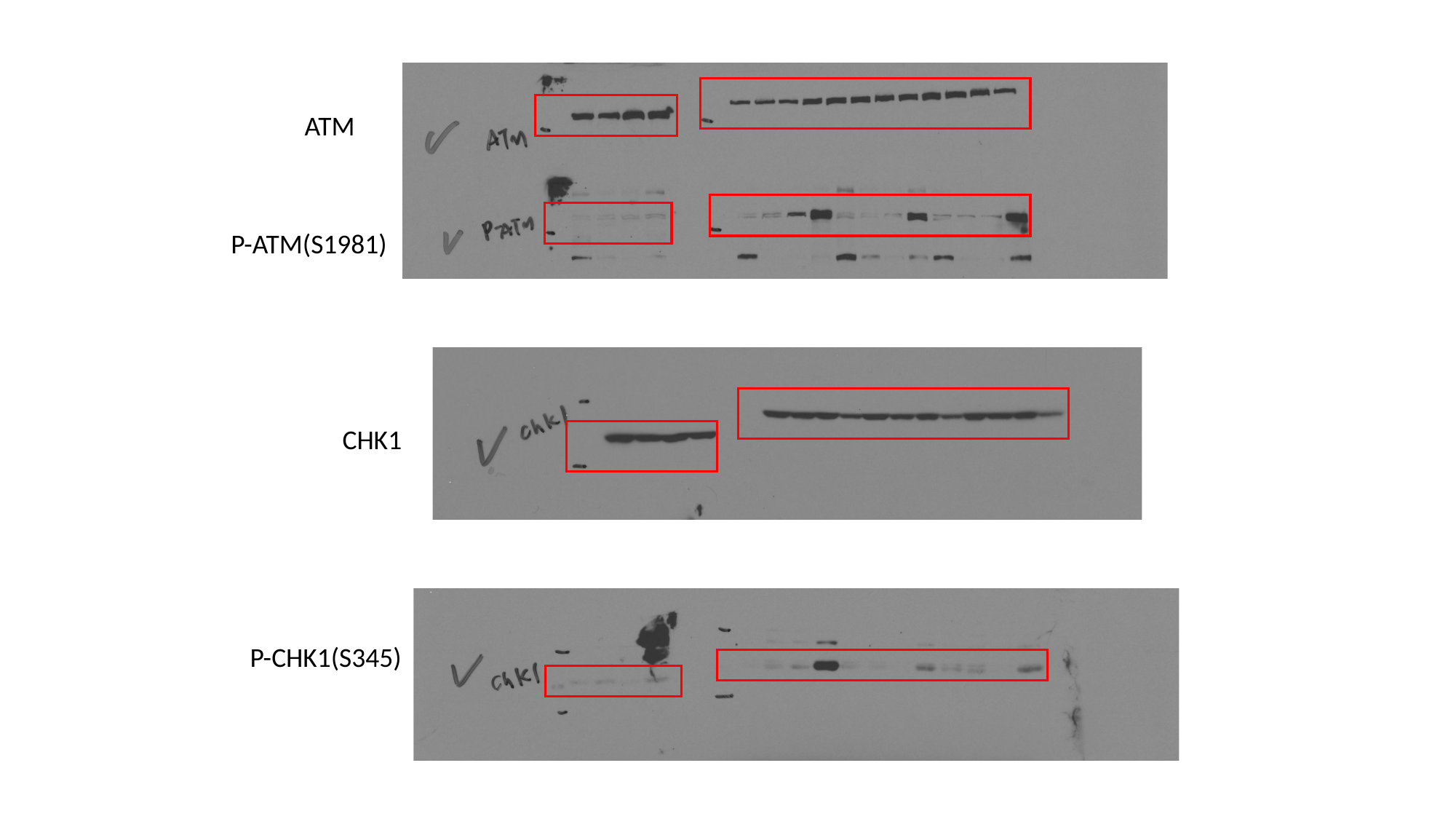

ATM
P-ATM(S1981)
CHK1
P-CHK1(S345)

## Slide 3
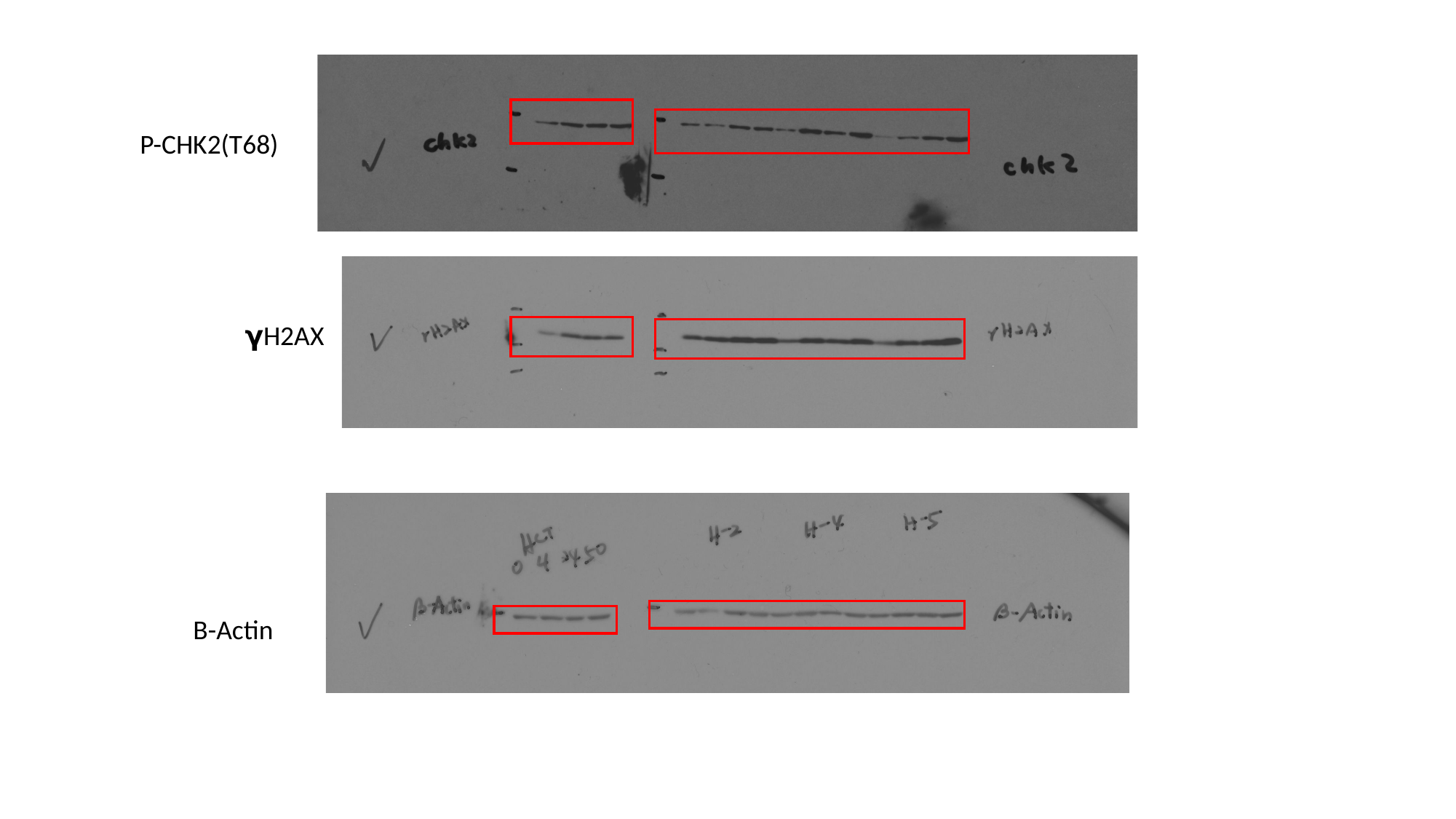

P-CHK2(T68)
𝝲H2AX
Β-Actin

Supplement: Figure 3—source data 2. [file elife-61797-fig3-data2.pptx]

## Slide 1
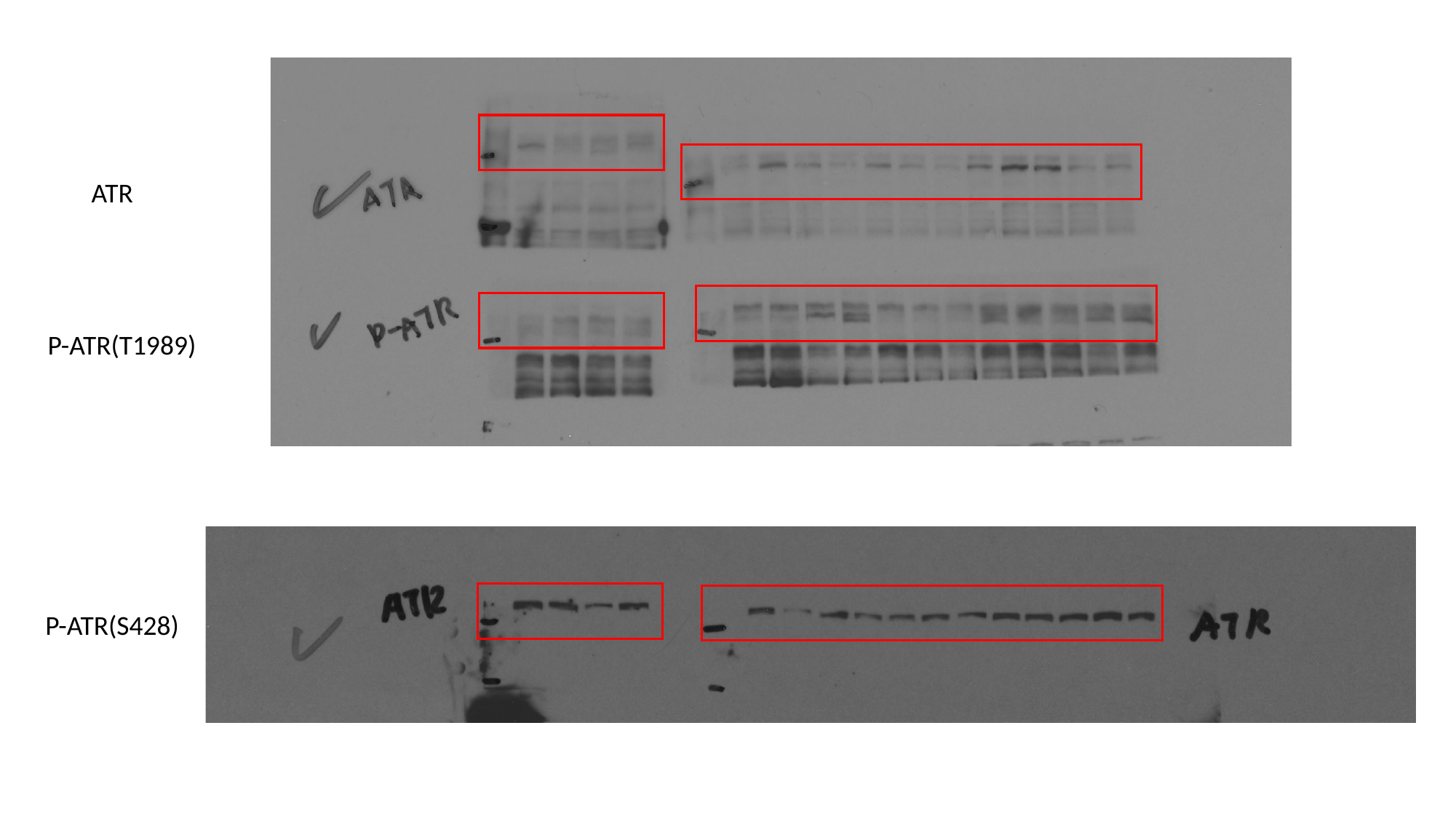

ATR
P-ATR(T1989)
P-ATR(S428)

Supplement: Figure 3—figure supplement 1—source data 1. [file elife-61797-fig3-figsupp1-data1.pptx]
