# Supplementary material for: The human origin recognition complex is essential for pre-RC assembly, mitosis, and maintenance of nuclear structure
Source: eLife. 2021 Feb 1;10:e61797. doi: 10.7554/eLife.61797 (PMC7877914; doi:10.7554/eLife.61797)
Supplement: Figure 3—source data 4. [file elife-61797-fig3-data4.pptx]

## Slide 1
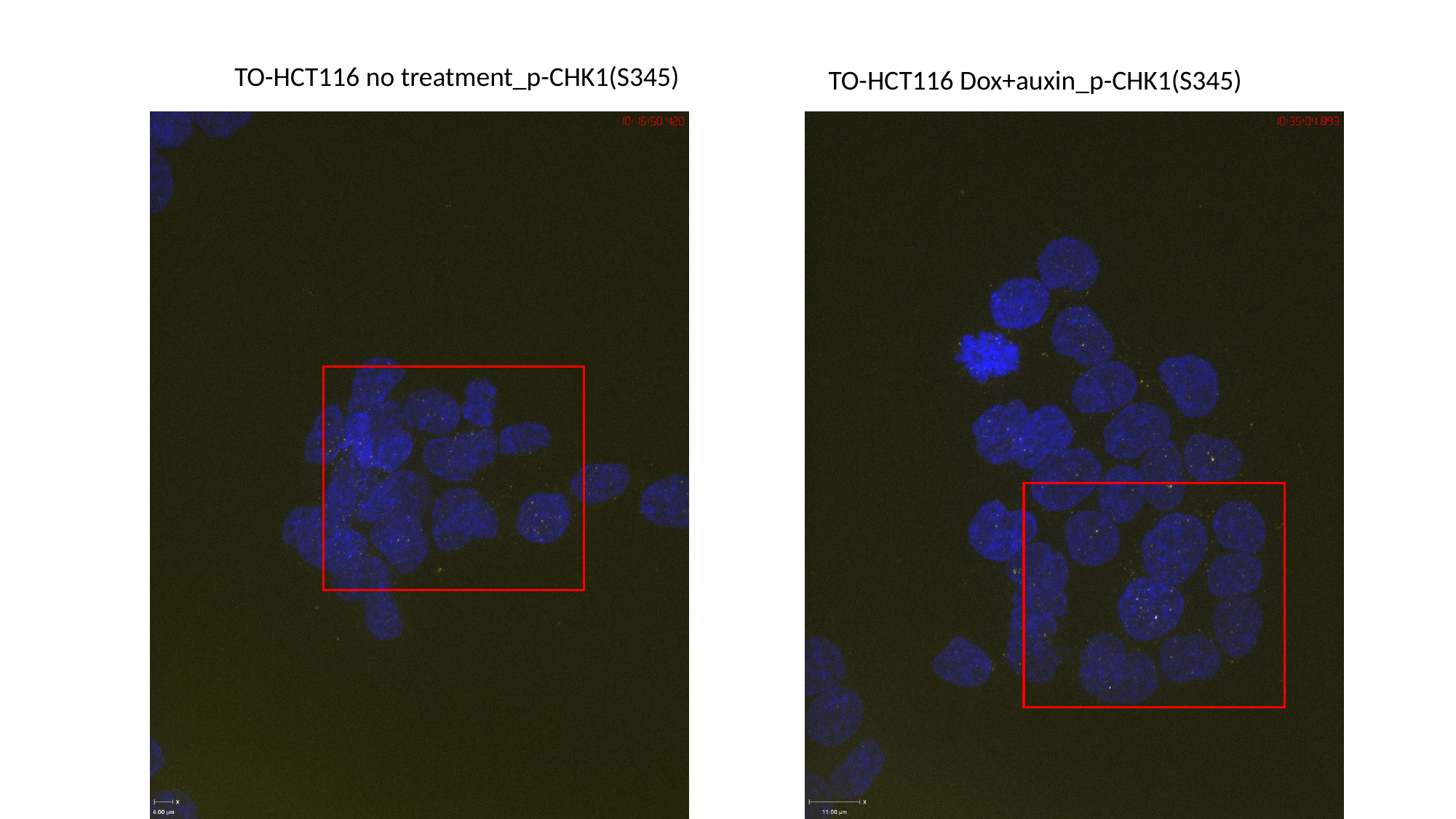

TO-HCT116 no treatment_p-CHK1(S345)
TO-HCT116 Dox+auxin_p-CHK1(S345)

## Slide 2
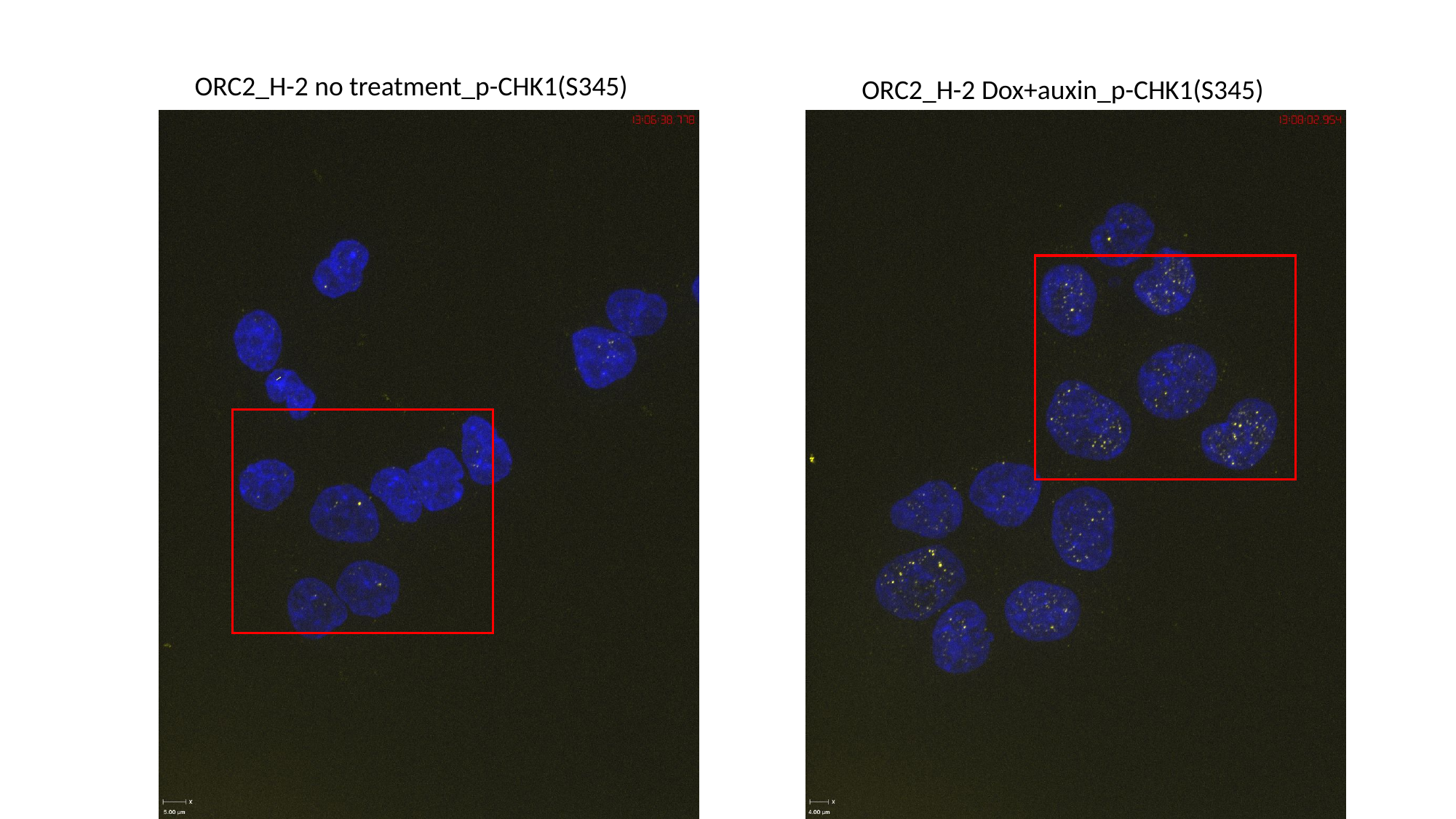

ORC2_H-2 no treatment_p-CHK1(S345)
ORC2_H-2 Dox+auxin_p-CHK1(S345)

## Slide 3
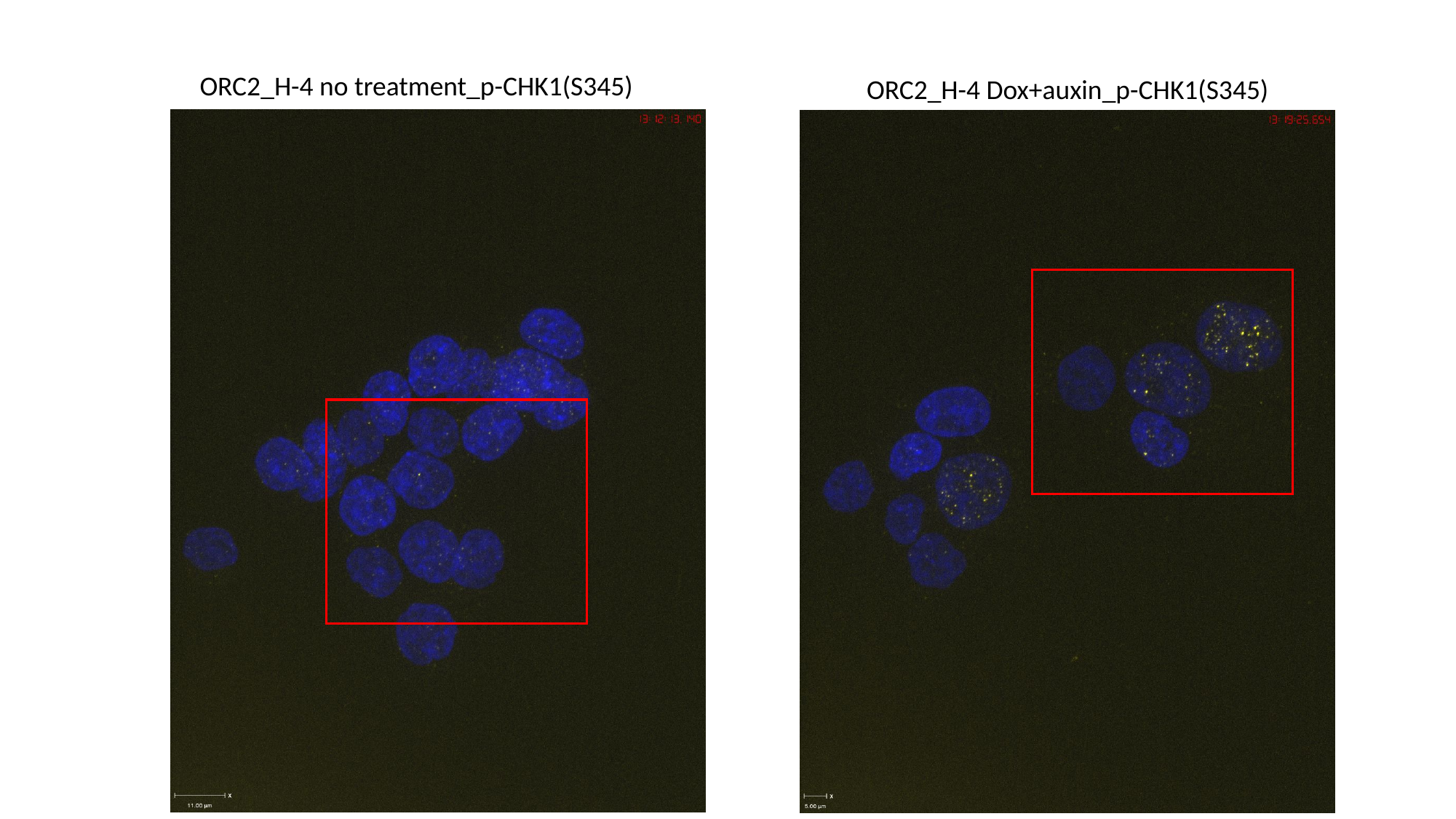

ORC2_H-4 no treatment_p-CHK1(S345)
ORC2_H-4 Dox+auxin_p-CHK1(S345)

## Slide 4
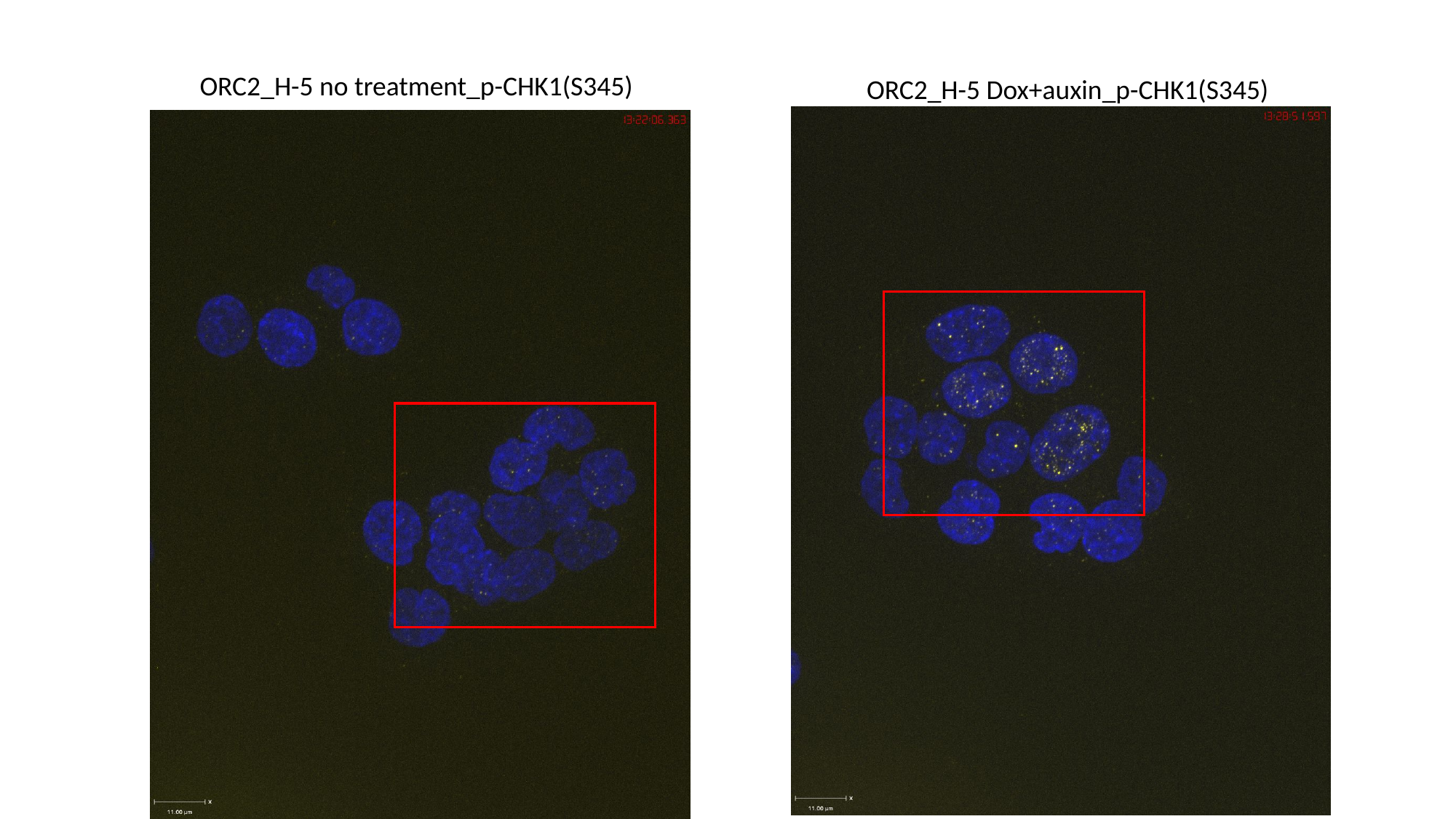

ORC2_H-5 no treatment_p-CHK1(S345)
ORC2_H-5 Dox+auxin_p-CHK1(S345)
