# Supplementary material for: The human origin recognition complex is essential for pre-RC assembly, mitosis, and maintenance of nuclear structure
Source: eLife. 2021 Feb 1;10:e61797. doi: 10.7554/eLife.61797 (PMC7877914; doi:10.7554/eLife.61797)
Supplement: Figure 4—source data 1. [file elife-61797-fig4-data1.pptx]

## Slide 1
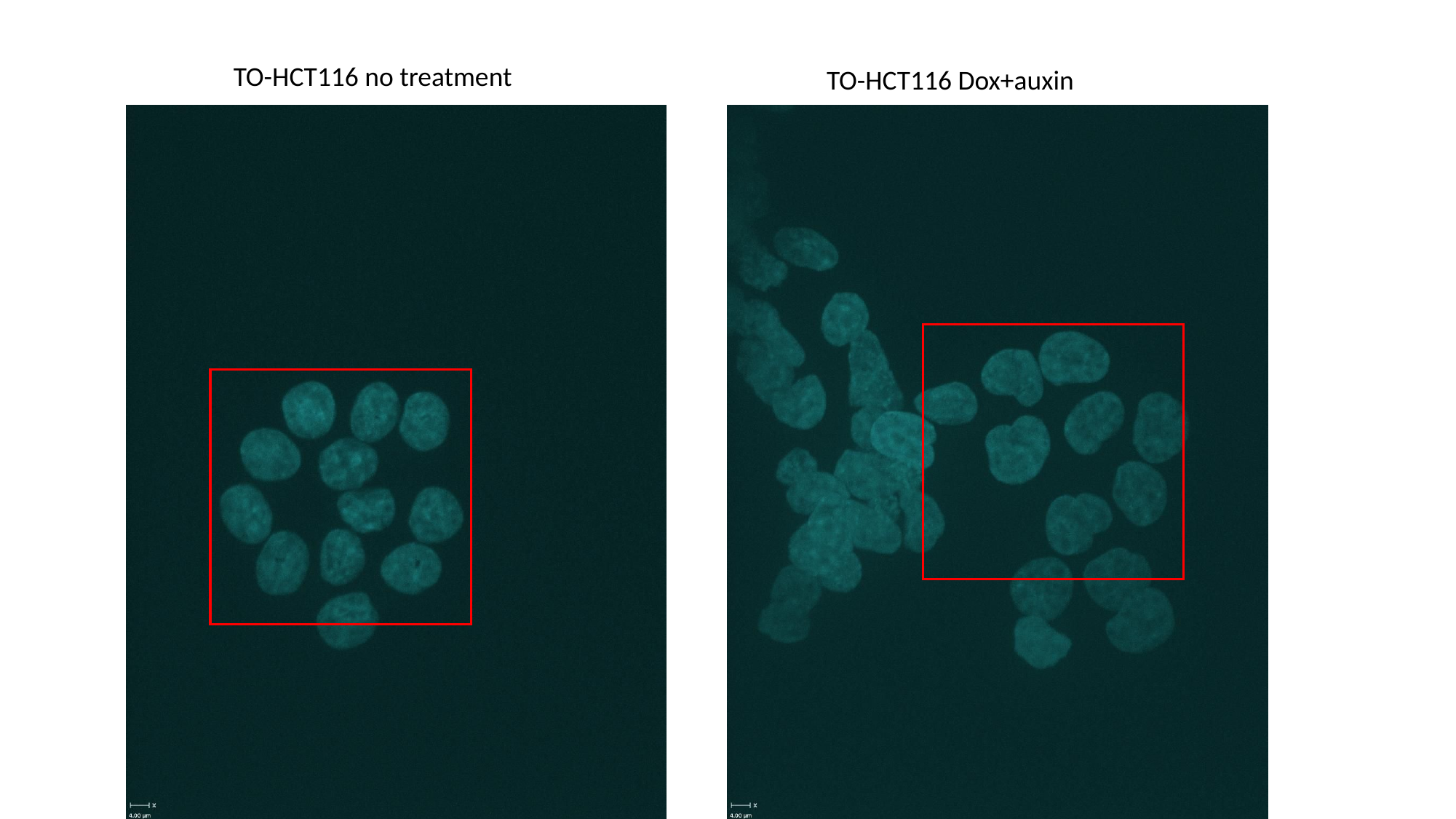

TO-HCT116 no treatment
TO-HCT116 Dox+auxin

## Slide 2
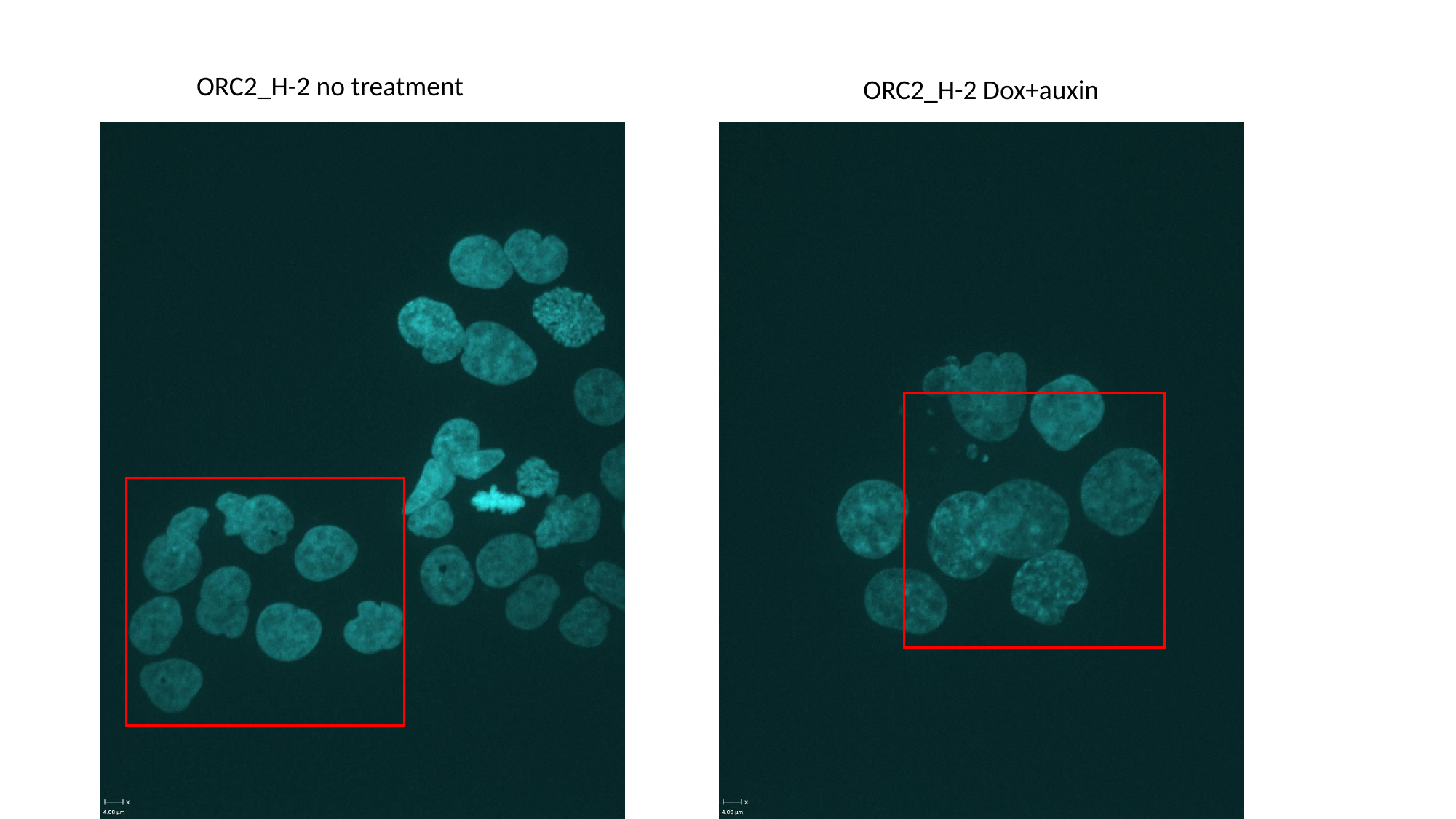

ORC2_H-2 no treatment
ORC2_H-2 Dox+auxin

## Slide 3
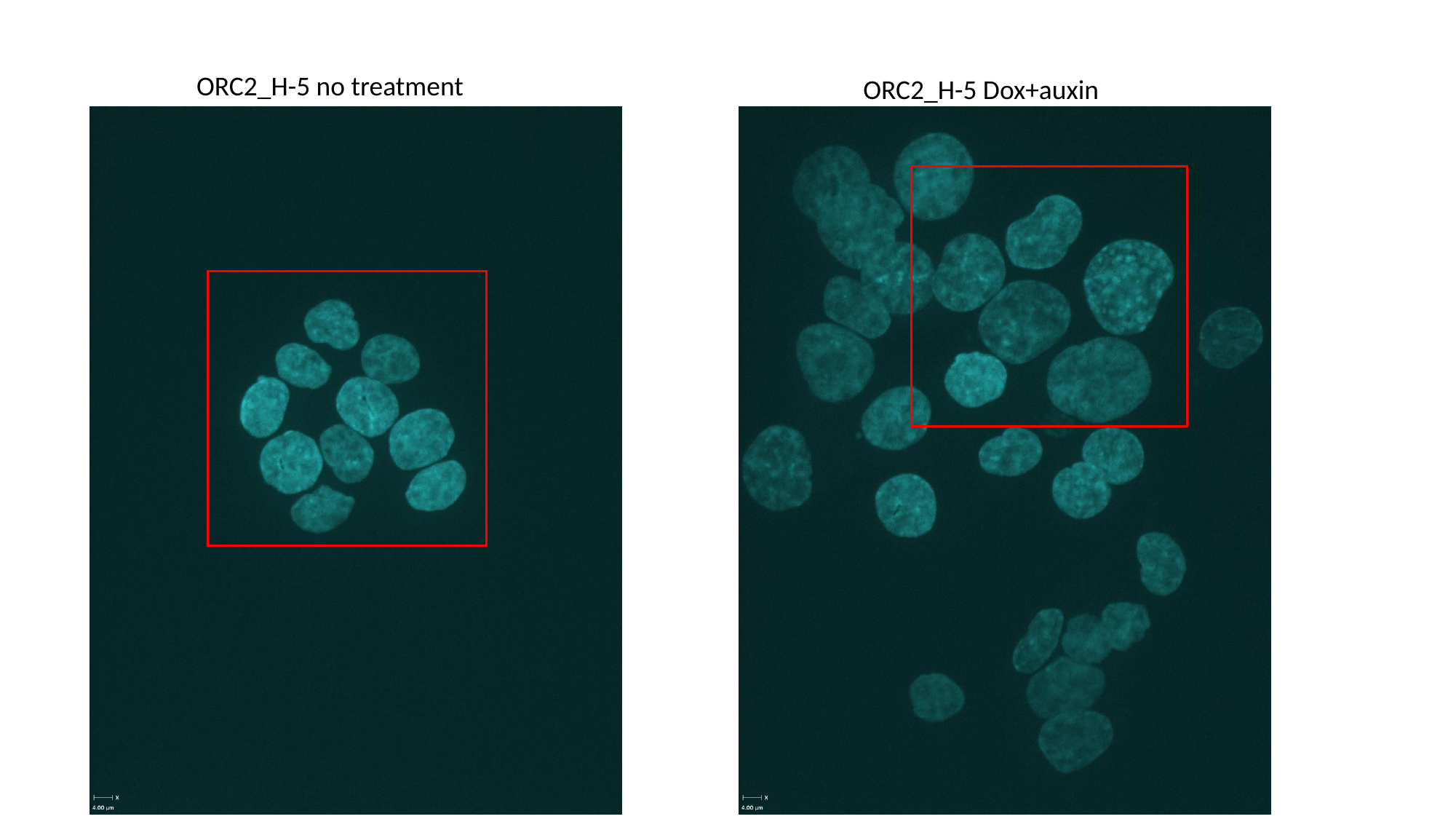

ORC2_H-5 no treatment
ORC2_H-5 Dox+auxin
