# Supplementary material for: The human origin recognition complex is essential for pre-RC assembly, mitosis, and maintenance of nuclear structure
Source: eLife. 2021 Feb 1;10:e61797. doi: 10.7554/eLife.61797 (PMC7877914; doi:10.7554/eLife.61797)
Supplement: Figure 4—source data 2. [file elife-61797-fig4-data2.docx]

Figure 4 -- source data 2. Nuclear volume (μm^3^)

| TO-HCT116  No treatment | ORC2_H-2  No treatment | ORC2_H-5  No treatment | TO-HCT116 Dox+auxin | ORC2_H-2 Dox+auxin | ORC2_H-5 Dox+auxin |
| --- | --- | --- | --- | --- | --- |
| 112.25 | 136.35 | 229.61 | 111.06 | 559.93 | 312.38 |
| 170.40 | 174.39 | 251.84 | 229.88 | 562.15 | 346.29 |
| 174.21 | 207.69 | 315.77 | 235.71 | 580.51 | 358.63 |
| 184.82 | 267.73 | 328.63 | 255.57 | 615.91 | 394.98 |
| 190.58 | 282.90 | 380.04 | 259.5 | 620.2 | 460.19 |
| 202.94 | 351.71 | 414.02 | 278.08 | 652.57 | 490.13 |
| 208.91 | 353.63 | 423.6 | 288.41 | 670.39 | 500.94 |
| 235.42 | 397.28 | 431.35 | 330.37 | 680.68 | 524.44 |
| 235.80 | 403.49 | 448.48 | 348.93 | 736.71 | 610.80 |
| 236.90 | 404.53 | 454.46 | 369.63 | 740.74 | 644.71 |
| 244.53 | 405.39 | 461.17 | 394.71 | 741.73 | 651.13 |
| 251.21 | 414.22 | 463.84 | 400.7 | 761.24 | 740.12 |
| 251.78 | 428.47 | 475.75 | 406.95 | 764.48 | 763.77 |
| 256.45 | 428.65 | 481.66 | 408.91 | 808.73 | 790.68 |
| 275.28 | 433.64 | 482.96 | 409.44 | 826.49 | 794.52 |
| 281.19 | 436.18 | 488.77 | 411.92 | 832.23 | 796.96 |
| 284.38 | 441.32 | 489.66 | 413.62 | 847.44 | 817.08 |
| 285.13 | 451.00 | 493.85 | 418.02 | 873.74 | 824.51 |
| 300.94 | 457.15 | 494.40 | 422.15 | 874.3 | 829.25 |
| 303.16 | 459.80 | 496.52 | 423.13 | 876.97 | 846.46 |
| 319.18 | 466.50 | 499.85 | 429.72 | 925.38 | 881.51 |
| 328.24 | 466.71 | 510.54 | 434.24 | 952.65 | 959.42 |
| 333.73 | 492.43 | 522.77 | 455.77 | 968.49 | 967.60 |
| 334.38 | 499.43 | 525.80 | 465.6 | 968.86 | 976.80 |
| 335.02 | 514.52 | 536.18 | 471.03 | 980.61 | 981.56 |
| 360.48 | 528.38 | 541.22 | 475.9 | 987.27 | 984.42 |
| 363.12 | 535.77 | 542.28 | 482.41 | 992.96 | 989.50 |
| 363.58 | 543.19 | 560.71 | 489.35 | 995.01 | 1009.87 |
| 364.46 | 581.03 | 570.09 | 489.95 | 1014.17 | 1038.68 |
| 365.84 | 589.95 | 570.23 | 490.11 | 1022.21 | 1049.45 |
| 367.89 | 590.55 | 589.28 | 499.04 | 1023.37 | 1054.16 |
| 368.39 | 613.93 | 603.91 | 500.48 | 1029.81 | 1069.84 |
| 375.42 | 622.45 | 607.36 | 503.36 | 1047.88 | 1079.77 |
| 376.49 | 632.33 | 607.94 | 515.71 | 1049.33 | 1082.55 |
| 377.93 | 637.57 | 611.78 | 515.81 | 1068.04 | 1130.82 |
| 383.81 | 640.05 | 621.3 | 519.92 | 1068.26 | 1139.78 |
| 386.95 | 641.37 | 626.69 | 523.68 | 1072.38 | 1150.68 |
| 390.51 | 650.47 | 642.08 | 523.90 | 1080.4 | 1151.74 |
| 391.07 | 654.73 | 677.13 | 526.77 | 1088.27 | 1154.27 |
| 404.08 | 657.77 | 679.67 | 530.33 | 1100.36 | 1160.05 |
| 406.31 | 664.33 | 686.46 | 533.82 | 1119.78 | 1160.82 |
| 418.24 | 674.46 | 691.89 | 535.26 | 1131.88 | 1194.98 |
| 418.87 | 678.56 | 693.57 | 542.52 | 1132.46 | 1223.93 |
| 420.76 | 683.57 | 695.07 | 543.52 | 1137.84 | 1234.91 |
| 426.83 | 697.45 | 703.32 | 547.12 | 1143 | 1321.79 |
| 427.84 | 712.87 | 705.94 | 559.09 | 1159.33 | 1330.10 |
| 432.19 | 724.10 | 706.83 | 576.61 | 1169.29 | 1334.32 |
| 467.58 | 734.87 | 726.76 | 582.6 | 1169.37 | 1342.80 |
| 478.80 | 762.38 | 734.48 | 583.11 | 1171.3 | 1538.04 |
| 479.09 | 772.38 | 745.52 | 587.23 | 1178.83 | 1670.44 |
| 482.93 | 843.36 | 760.96 | 589.49 | 1206.49 | 1821.62 |
| 483.50 | 941.57 | 777.33 | 594.22 | 1209.05 | 1995.72 |
| 498.98 |  | 778.23 | 623.38 | 1218.92 | 2644.16 |
| 501.87 |  | 783.33 | 630.67 | 1222.17 | 3278.82 |
| 501.99 |  | 787.97 | 655.10 | 1223.06 |  |
| 502.70 |  | 806.19 | 655.84 | 1223.92 |  |
| 519.02 |  | 823.51 | 671.16 | 1226.12 |  |
| 522.24 |  | 824.26 | 681.74 | 1233.66 |  |
| 522.37 |  | 829.33 | 683.27 | 1237.24 |  |
| 523.80 |  | 861.38 | 691.09 | 1249.34 |  |
| 528.73 |  | 914.05 | 720.47 | 1259.23 |  |
| 540.06 |  | 1148.91 | 725.36 | 1278.73 |  |
| 565.55 |  | 1386.1 | 790.72 | 1284.81 |  |
| 568.02 |  |  | 807.13 | 1298.03 |  |
| 576.53 |  |  | 905.11 | 1300.92 |  |
| 592.04 |  |  | 934.06 | 1302.89 |  |
| 592.64 |  |  |  | 1303.79 |  |
| 597.51 |  |  |  | 1314.63 |  |
| 626.74 |  |  |  | 1316.48 |  |
| 647.29 |  |  |  | 1327.67 |  |
| 664.33 |  |  |  | 1331.55 |  |
| 668.60 |  |  |  | 1341.59 |  |
| 671.93 |  |  |  | 1355.57 |  |
| 718.89 |  |  |  | 1359.38 |  |
| 754.79 |  |  |  | 1382.56 |  |
| 772.38 |  |  |  | 1386.26 |  |
| 843.36 |  |  |  | 1388.68 |  |
|  |  |  |  | 1388.97 |  |
|  |  |  |  | 1409.19 |  |
|  |  |  |  | 1415.65 |  |
|  |  |  |  | 1428.88 |  |
|  |  |  |  | 1429.00 |  |
|  |  |  |  | 1436.74 |  |
|  |  |  |  | 1450.97 |  |
|  |  |  |  | 1453.85 |  |
|  |  |  |  | 1468.35 |  |
|  |  |  |  | 1468.38 |  |
|  |  |  |  | 1488.84 |  |
|  |  |  |  | 1490.39 |  |
|  |  |  |  | 1496.16 |  |
|  |  |  |  | 1502.86 |  |
|  |  |  |  | 1503.53 |  |
|  |  |  |  | 1517.54 |  |
|  |  |  |  | 1533.23 |  |
|  |  |  |  | 1534.50 |  |
|  |  |  |  | 1542.91 |  |
|  |  |  |  | 1552.72 |  |
|  |  |  |  | 1562.29 |  |
|  |  |  |  | 1589.13 |  |
|  |  |  |  | 1592.03 |  |
|  |  |  |  | 1657.25 |  |
|  |  |  |  | 1657.92 |  |
|  |  |  |  | 1678.00 |  |
|  |  |  |  | 1742.48 |  |
|  |  |  |  | 1747.98 |  |
|  |  |  |  | 1756.04 |  |
|  |  |  |  | 2041.49 |  |
|  |  |  |  | 2371.83 |  |
|  |  |  |  | 2483.91 |  |
|  |  |  |  | 2599.29 |  |
