# Supplementary material for: The human origin recognition complex is essential for pre-RC assembly, mitosis, and maintenance of nuclear structure
Source: eLife. 2021 Feb 1;10:e61797. doi: 10.7554/eLife.61797 (PMC7877914; doi:10.7554/eLife.61797)
Supplement: Figure 7—source data 1. [file elife-61797-fig7-data1.docx]

Figure 7 – source data 1. P-H3S10 pos/neg cells percentage (of total population) in the fraction of G2/M cells

|  | positive for p-H3S10 (%) | | | Negative for p-H3S10 (%) | | |
| --- | --- | --- | --- | --- | --- | --- |
|  | replicate 1 | replicate 2 | replicate 3 | replicate 1 | replicate 2 | replicate 3 |
| TO-HCT116 | 5.0025 | 4.3878 | 3.8304 | 29.498 | 26.5122 | 24.97 |
| TO-HCT116 dox | 4.216 | 4.161 | 3.4272 | 20.584 | 14.839 | 16.973 |
| TO-HCT116 28hr | 2.533 | 2.0584 | 2.4254 | 14.467 | 14.5416 | 15.675 |
| TO-HCT116 50hr | 3.7412 | 4.047 | 3.9294 | 15.059 | 17.253 | 18.271 |
| ORC2_H-2 | 1.34236 | 1.39826 | 1.9494 | 13.458 | 13.70174 | 15.151 |
| ORC2_H-2 dox | 1.3794 | 1.03768 | 1.10223 | 10.721 | 8.48232 | 8.8278 |
| ORC2_H-2 28hr | 0.72962 | 0.89298 | 0.735 | 37.47 | 36.00702 | 34.265 |
| ORC2_H-2 50hr | 0.89936 | 1.12161 | 0.82921 | 79.401 | 75.17839 | 81.271 |
