# Supplementary material for: The human origin recognition complex is essential for pre-RC assembly, mitosis, and maintenance of nuclear structure
Source: eLife. 2021 Feb 1;10:e61797. doi: 10.7554/eLife.61797 (PMC7877914; doi:10.7554/eLife.61797)
Supplement: Figure 7—source data 4. [file elife-61797-fig7-data4.pptx]

## Slide 1
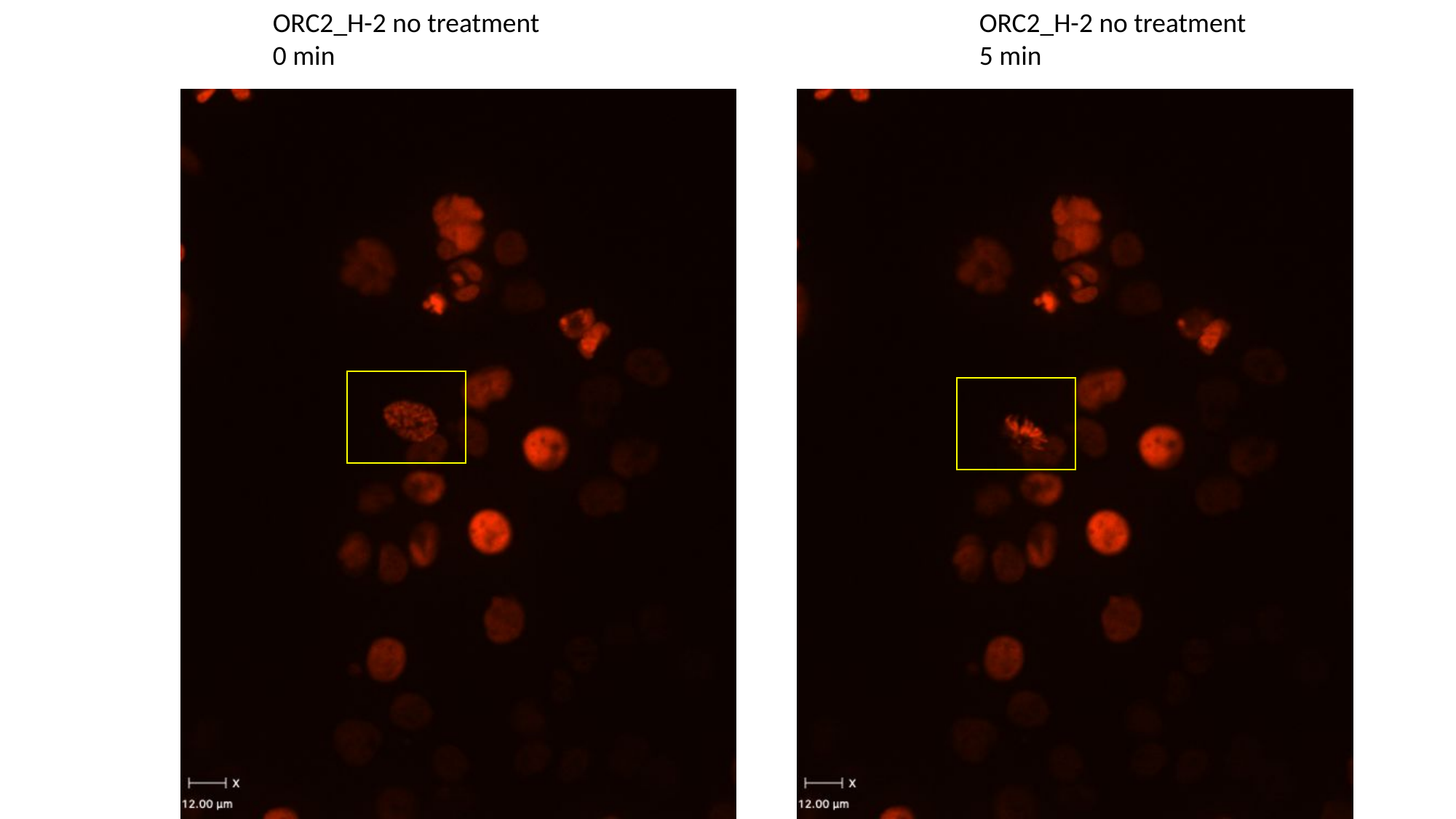

ORC2_H-2 no treatment
0 min
ORC2_H-2 no treatment
5 min

## Slide 2
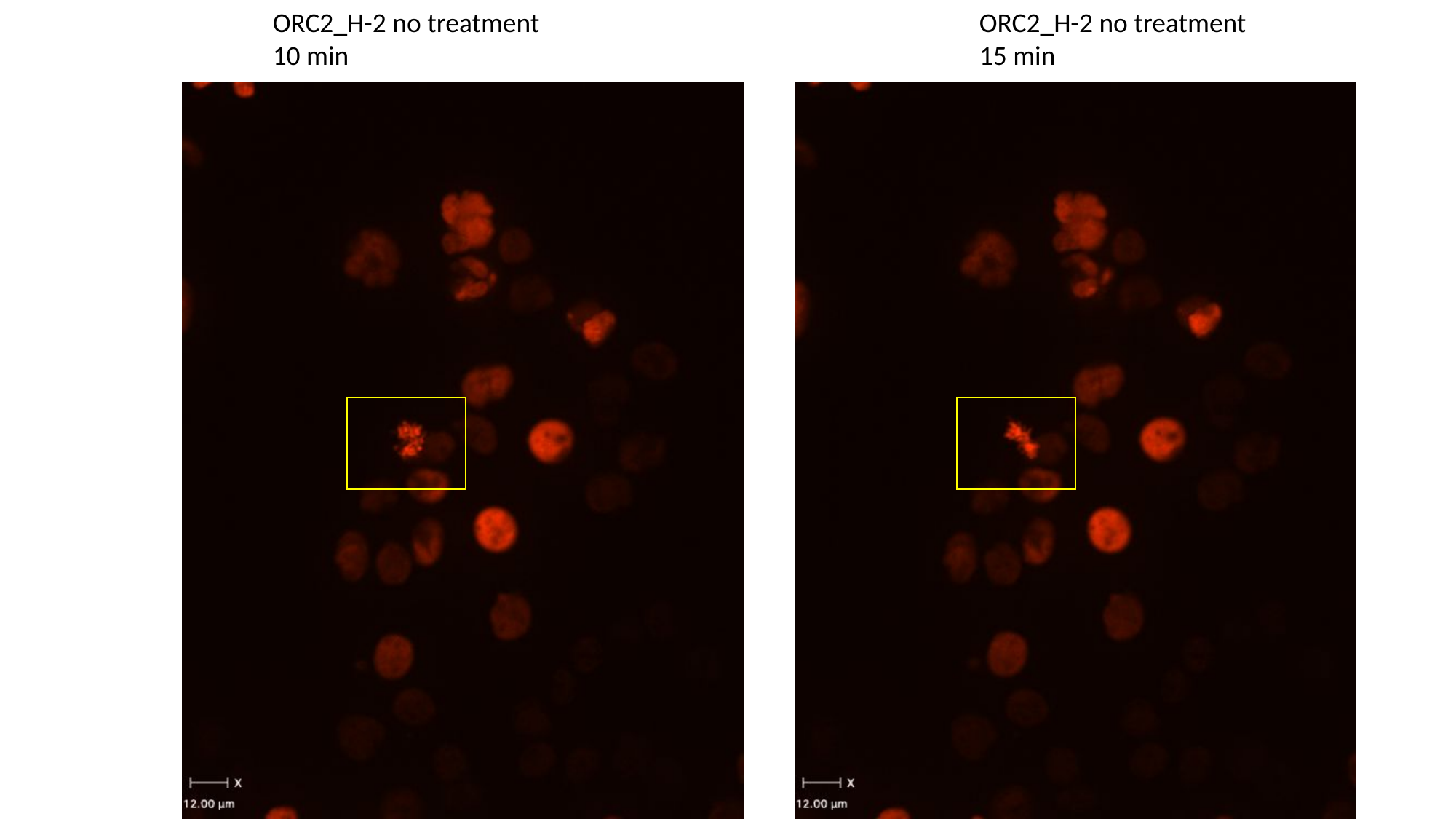

ORC2_H-2 no treatment
10 min
ORC2_H-2 no treatment
15 min

## Slide 3
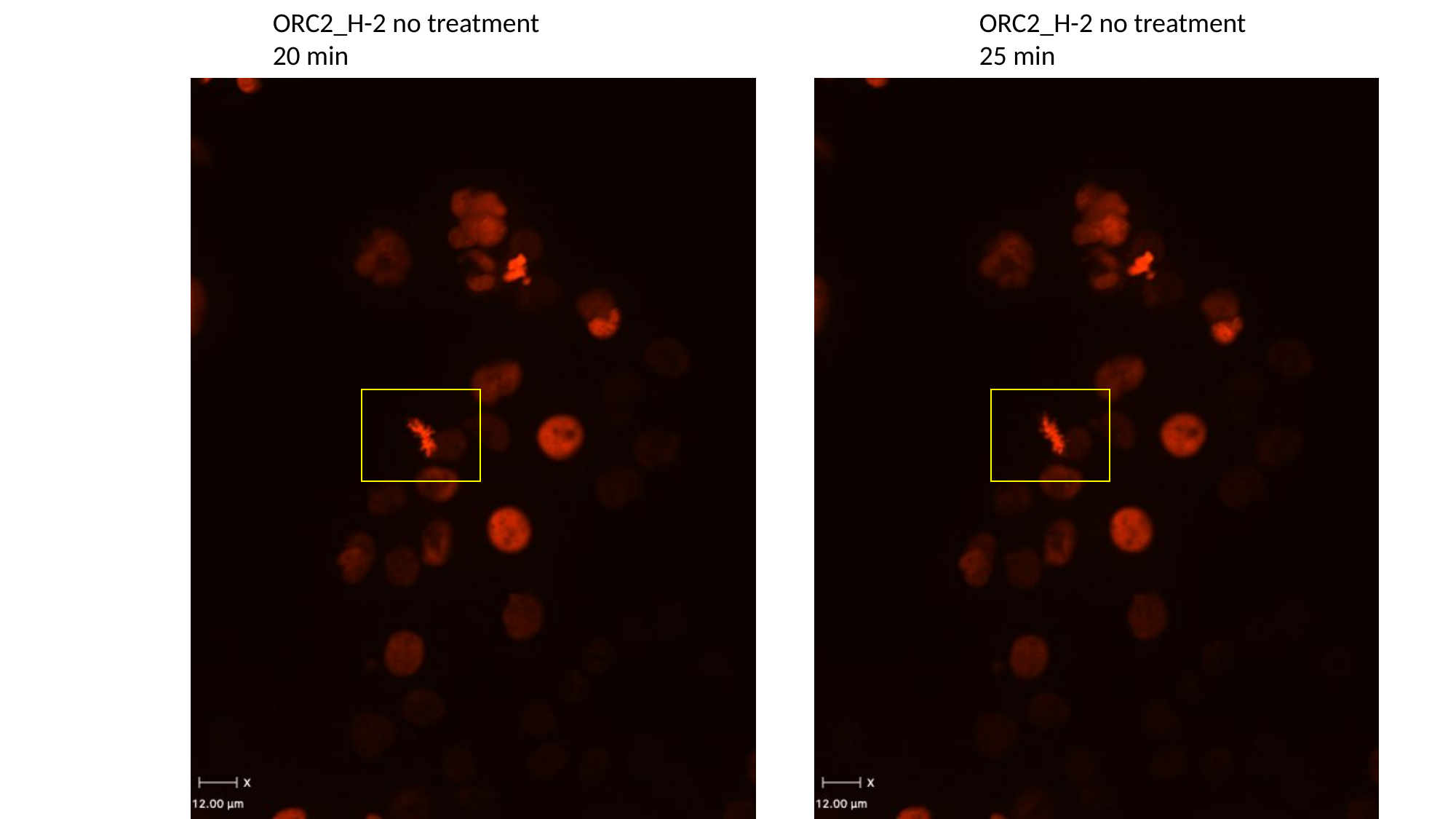

ORC2_H-2 no treatment
20 min
ORC2_H-2 no treatment
25 min

## Slide 4
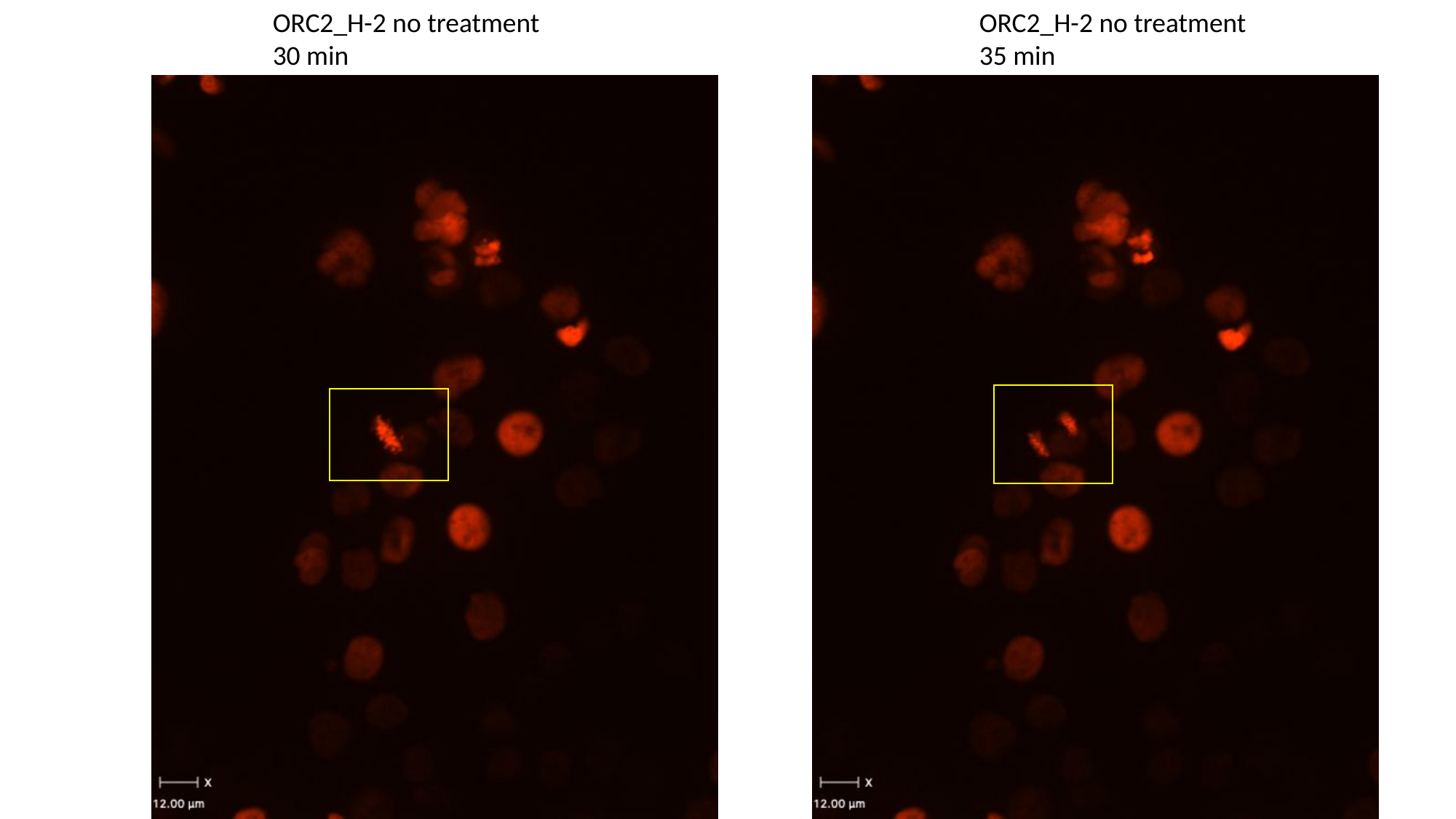

ORC2_H-2 no treatment
30 min
ORC2_H-2 no treatment
35 min
