# Supplementary material for: The human origin recognition complex is essential for pre-RC assembly, mitosis, and maintenance of nuclear structure
Source: eLife. 2021 Feb 1;10:e61797. doi: 10.7554/eLife.61797 (PMC7877914; doi:10.7554/eLife.61797)
Supplement: Figure 7—source data 5. [file elife-61797-fig7-data5.pptx]

## Slide 1
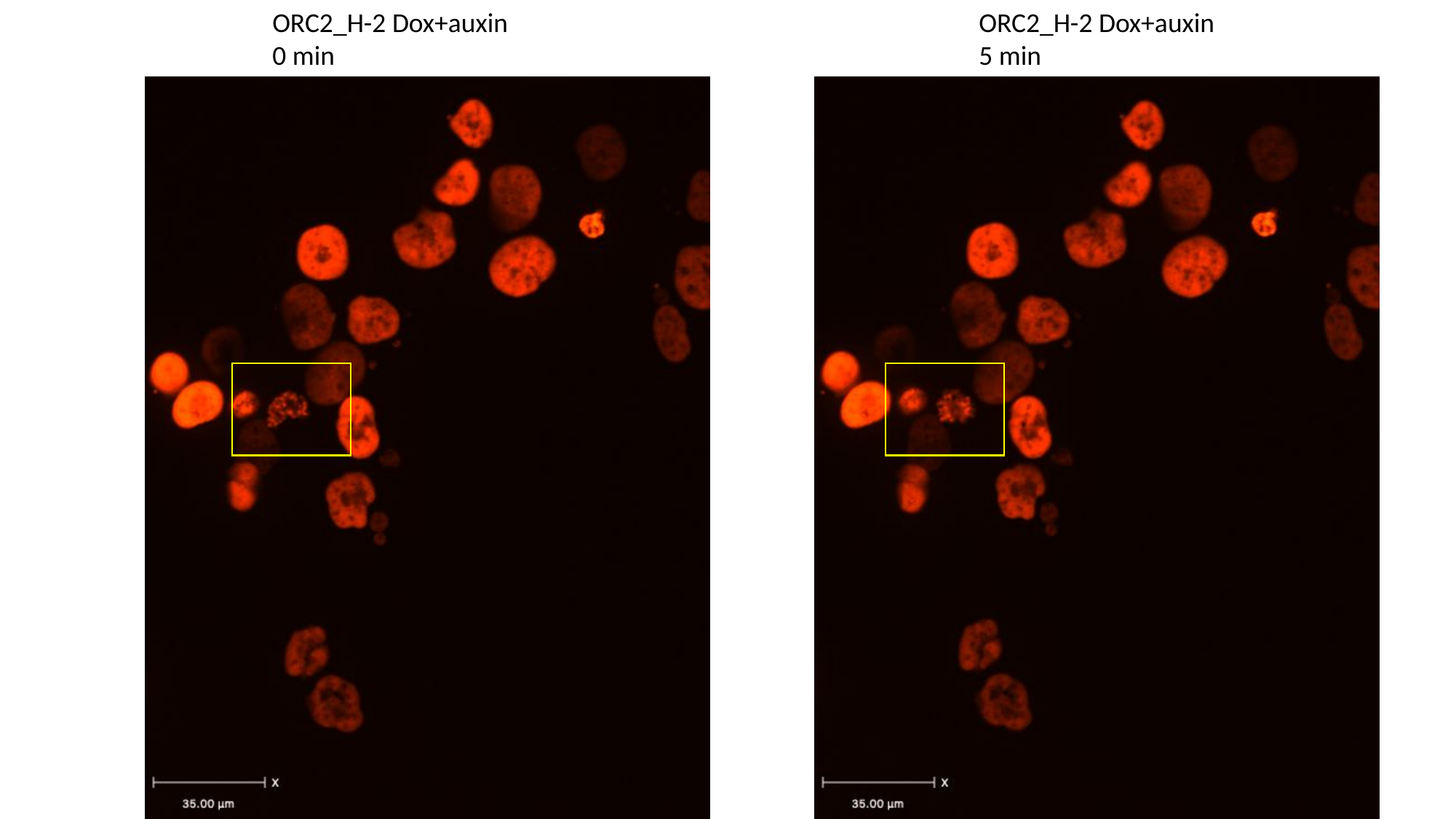

ORC2_H-2 Dox+auxin
0 min
ORC2_H-2 Dox+auxin
5 min

## Slide 2
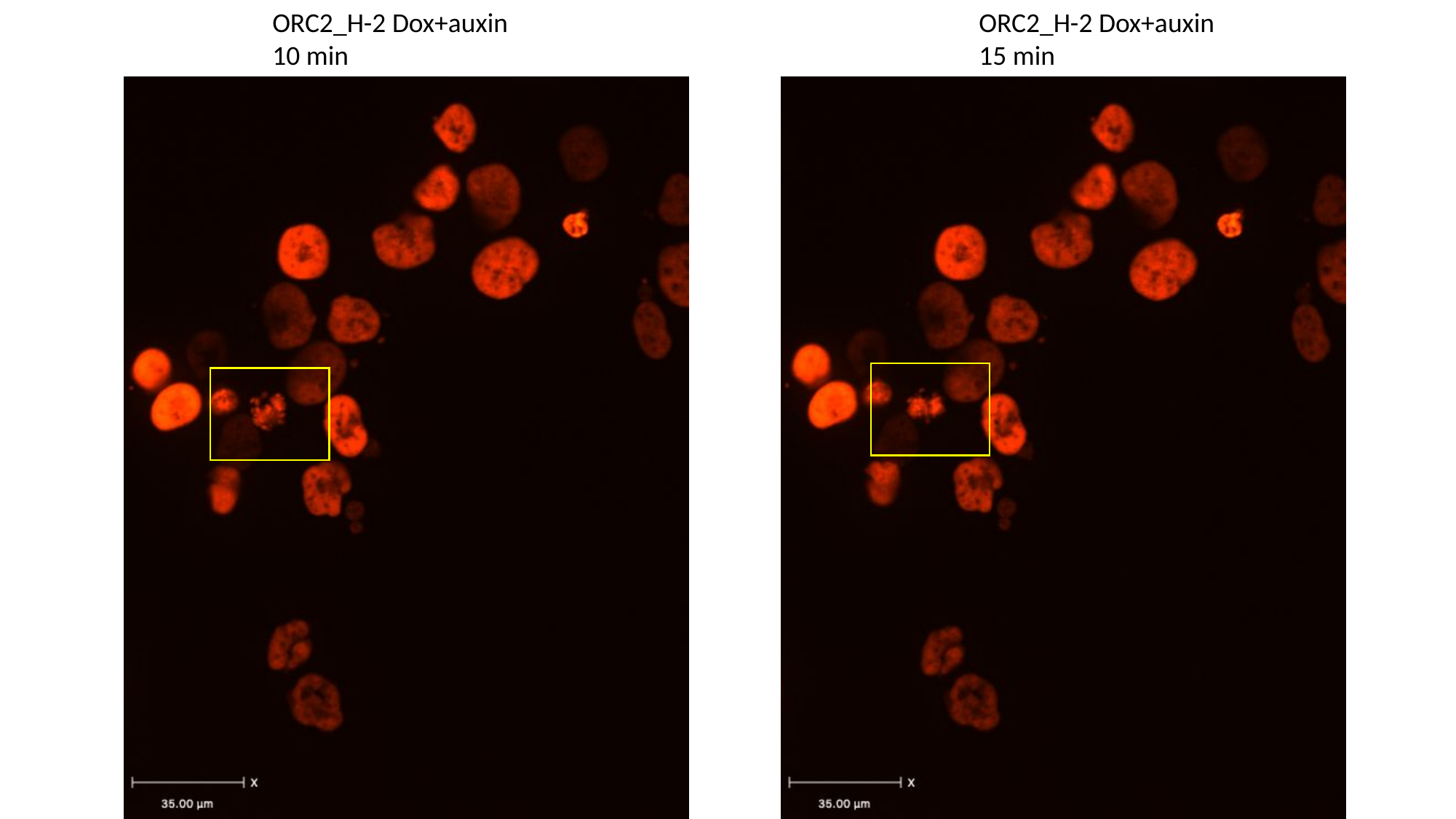

ORC2_H-2 Dox+auxin
10 min
ORC2_H-2 Dox+auxin
15 min

## Slide 3
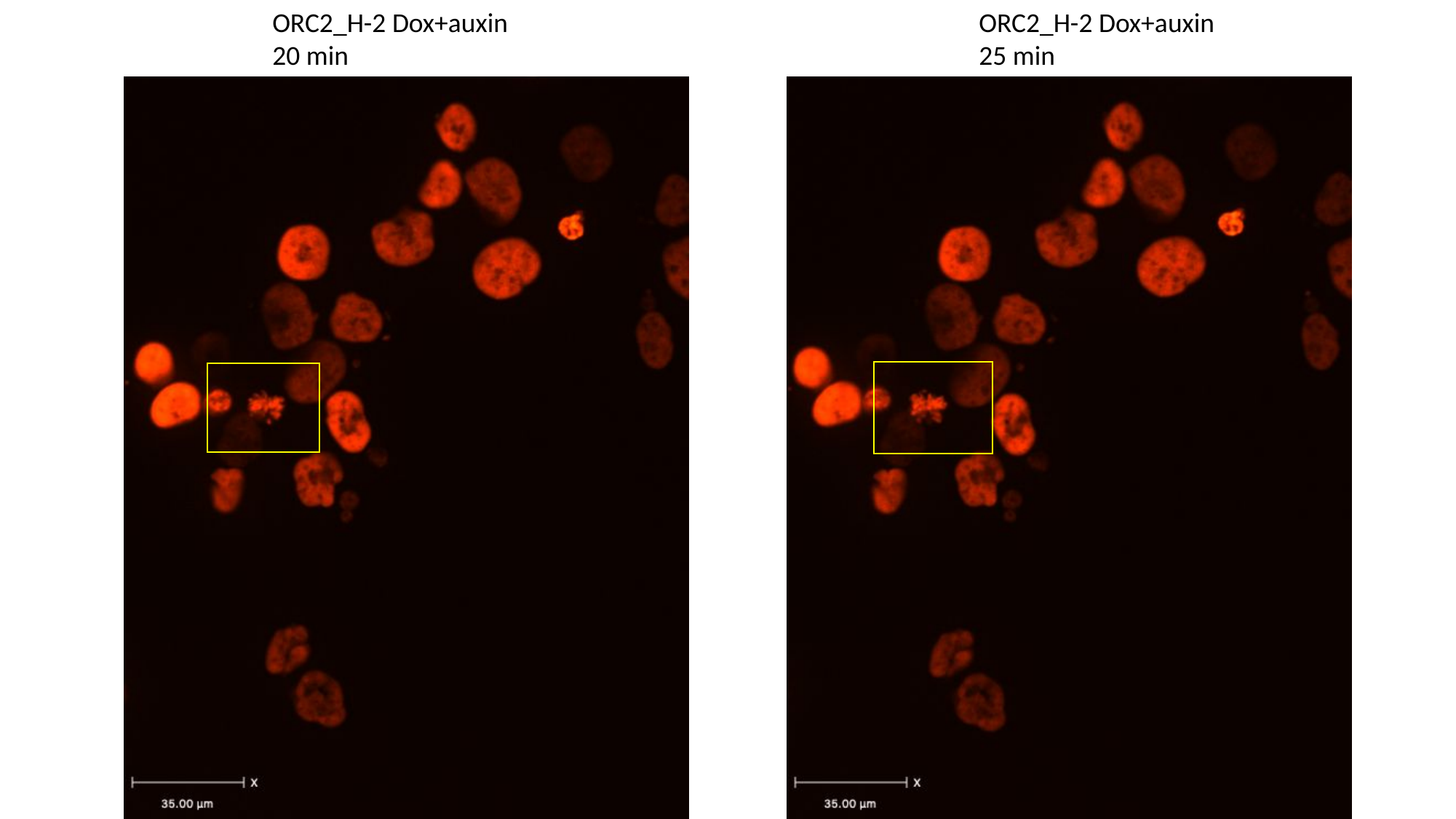

ORC2_H-2 Dox+auxin
20 min
ORC2_H-2 Dox+auxin
25 min

## Slide 4
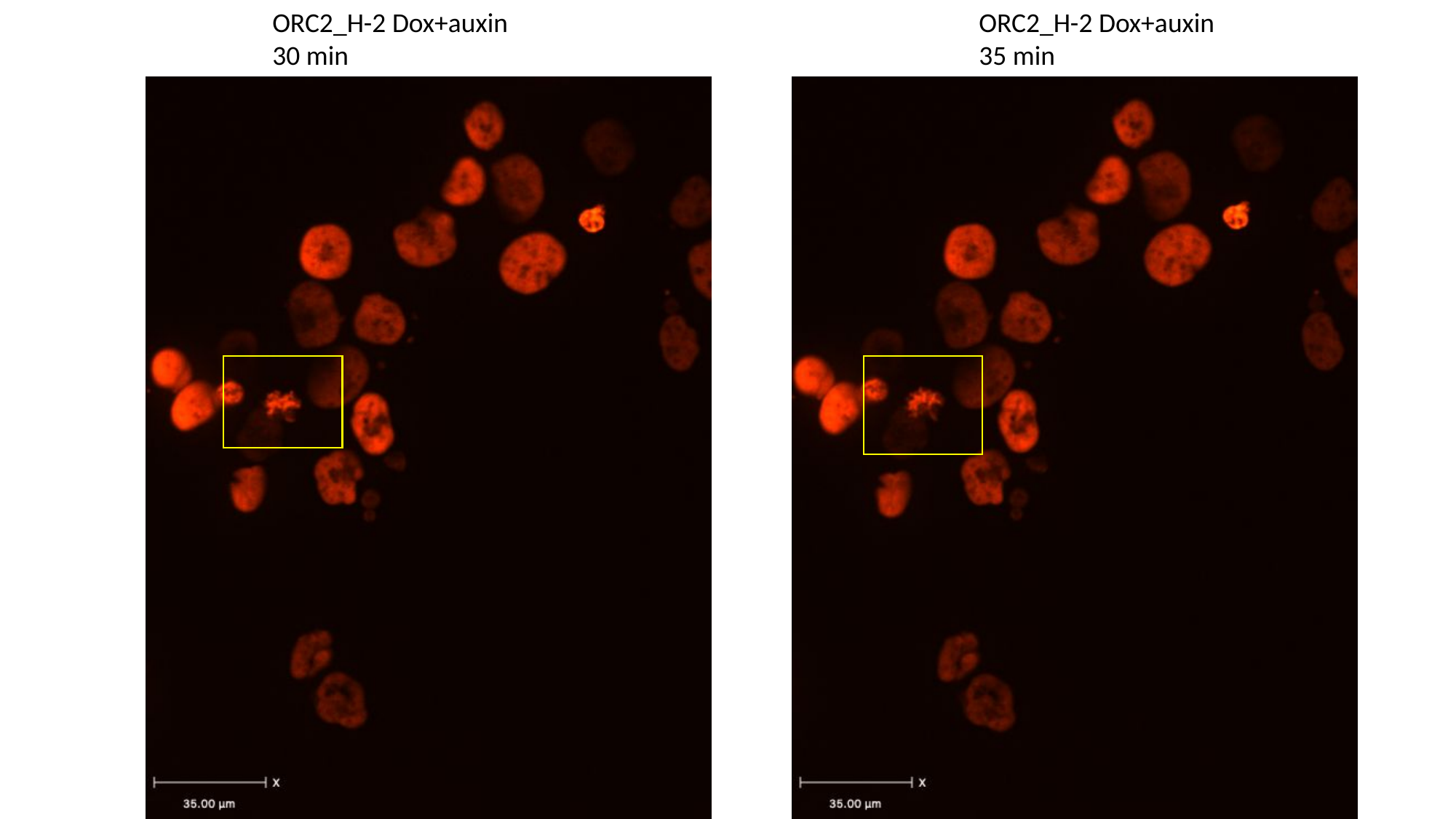

ORC2_H-2 Dox+auxin
30 min
ORC2_H-2 Dox+auxin
35 min

## Slide 5
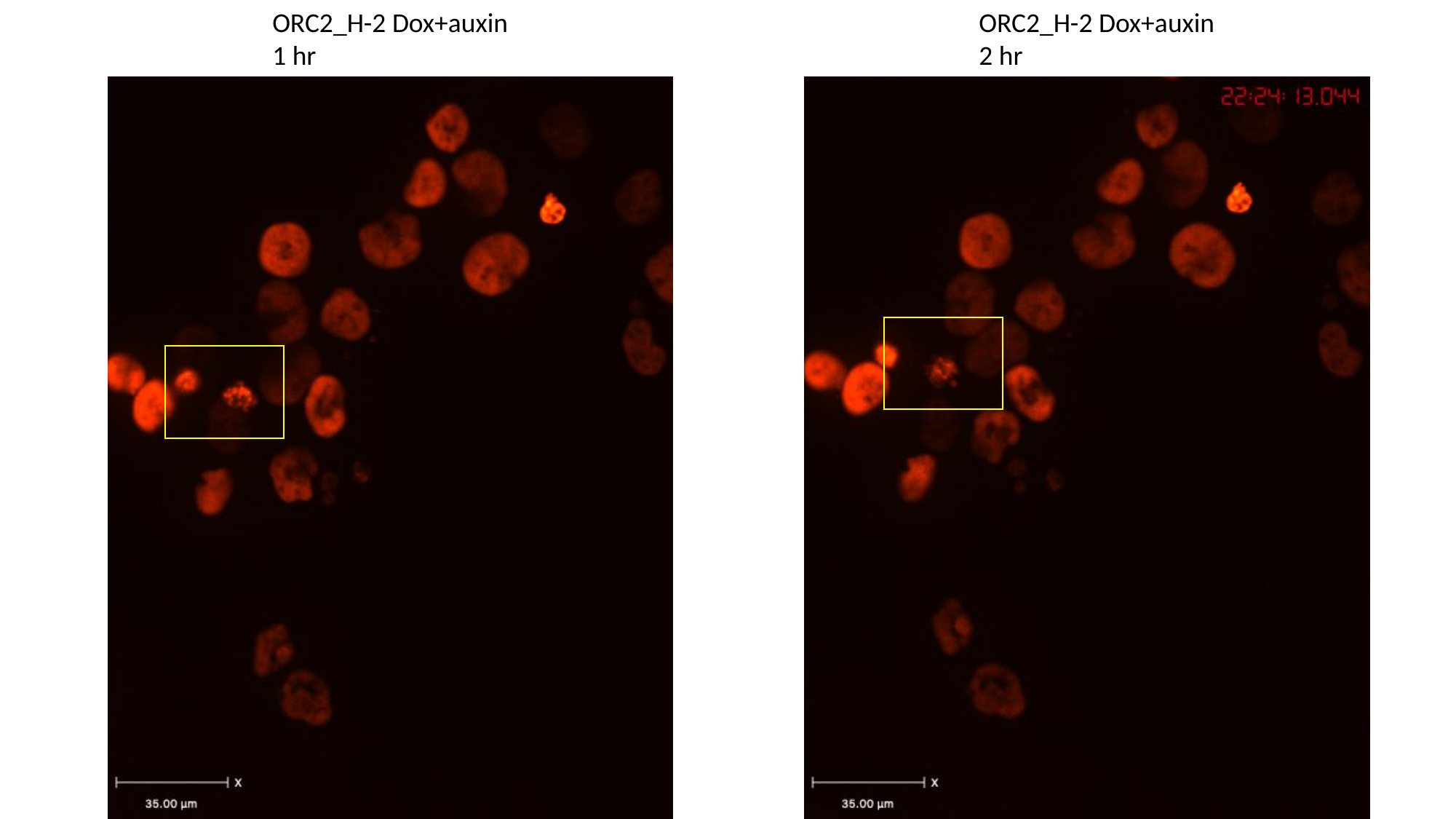

ORC2_H-2 Dox+auxin
1 hr
ORC2_H-2 Dox+auxin
2 hr

## Slide 6
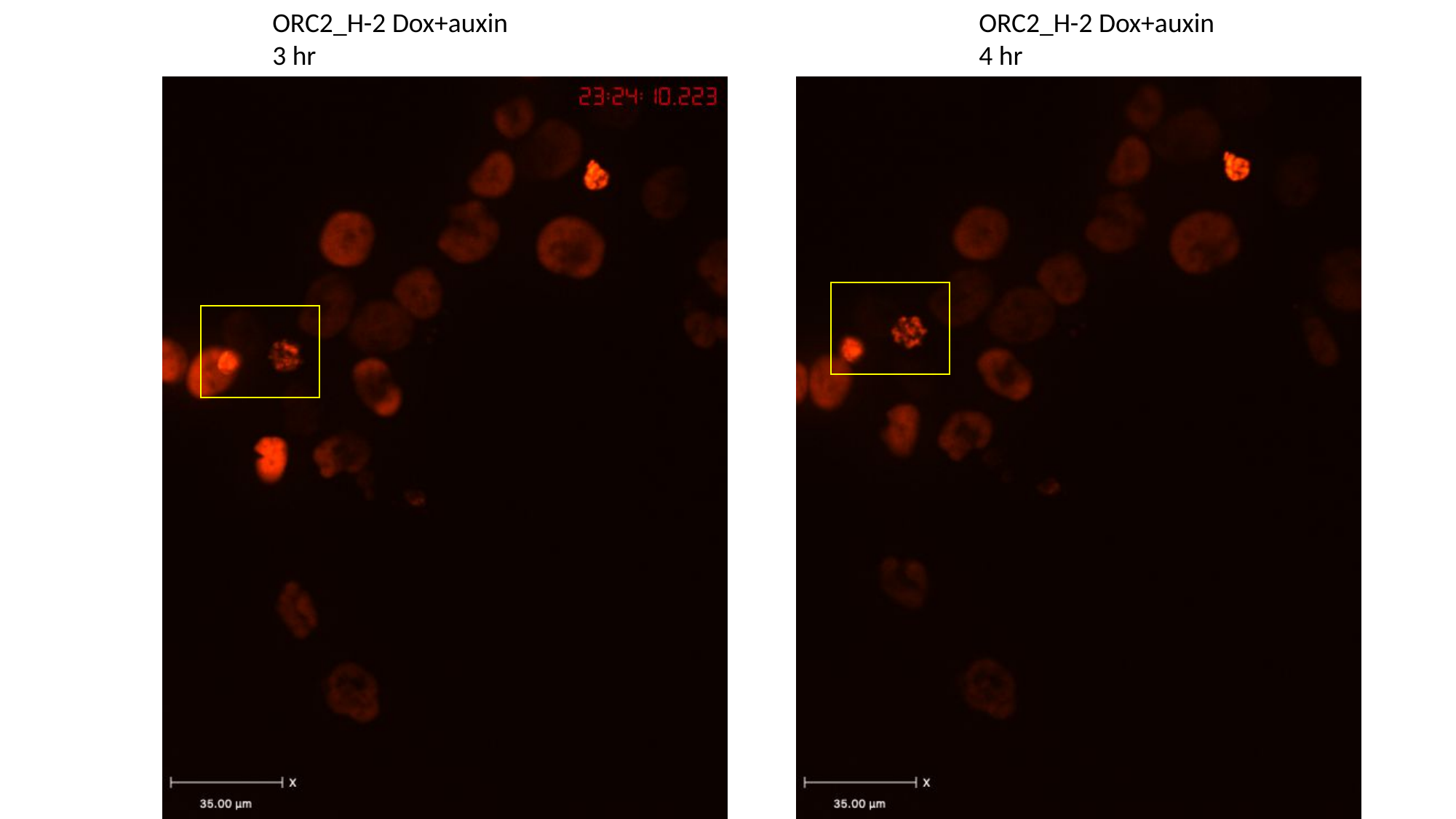

ORC2_H-2 Dox+auxin
3 hr
ORC2_H-2 Dox+auxin
4 hr

## Slide 7
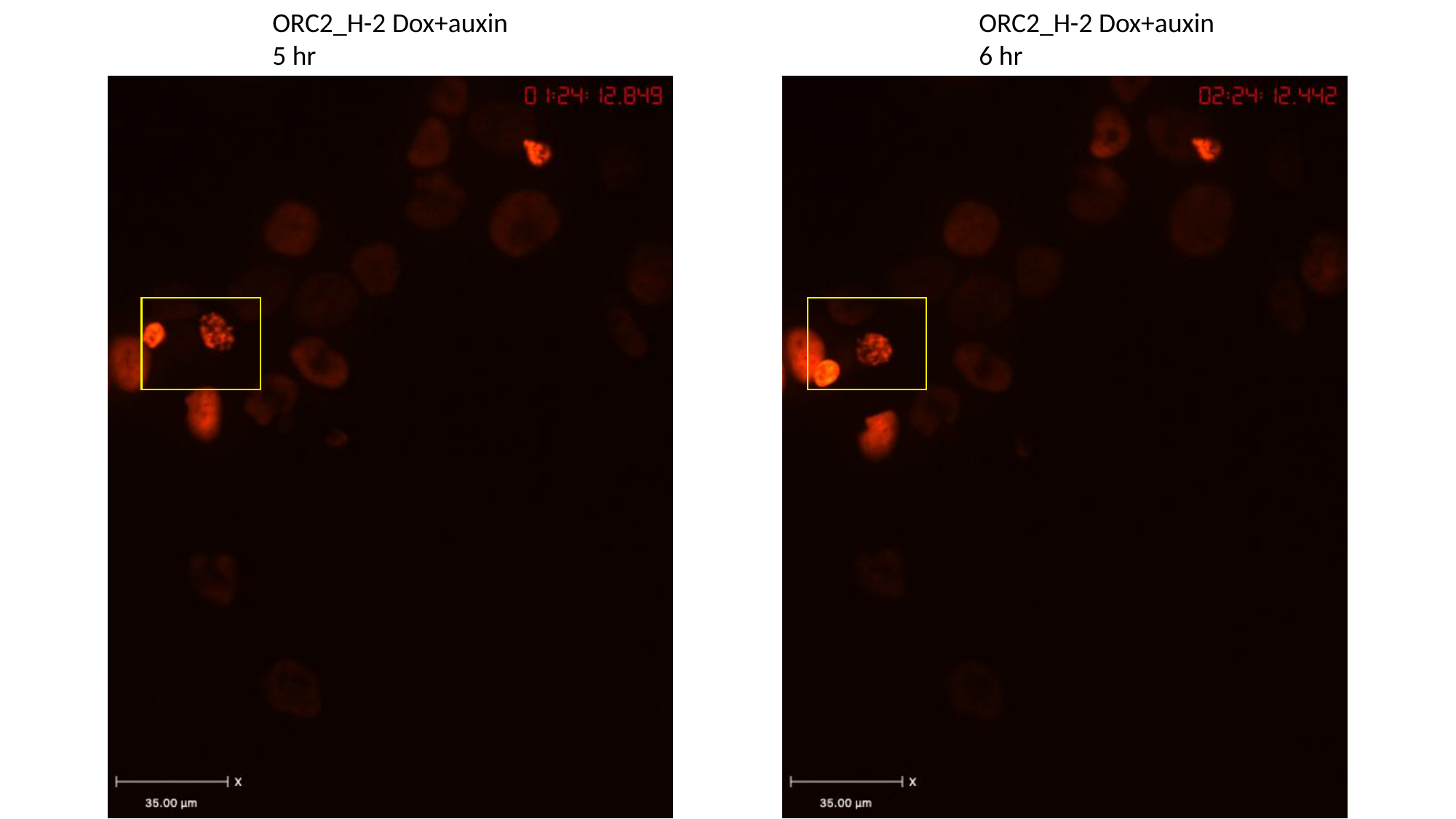

ORC2_H-2 Dox+auxin
5 hr
ORC2_H-2 Dox+auxin
6 hr

## Slide 8
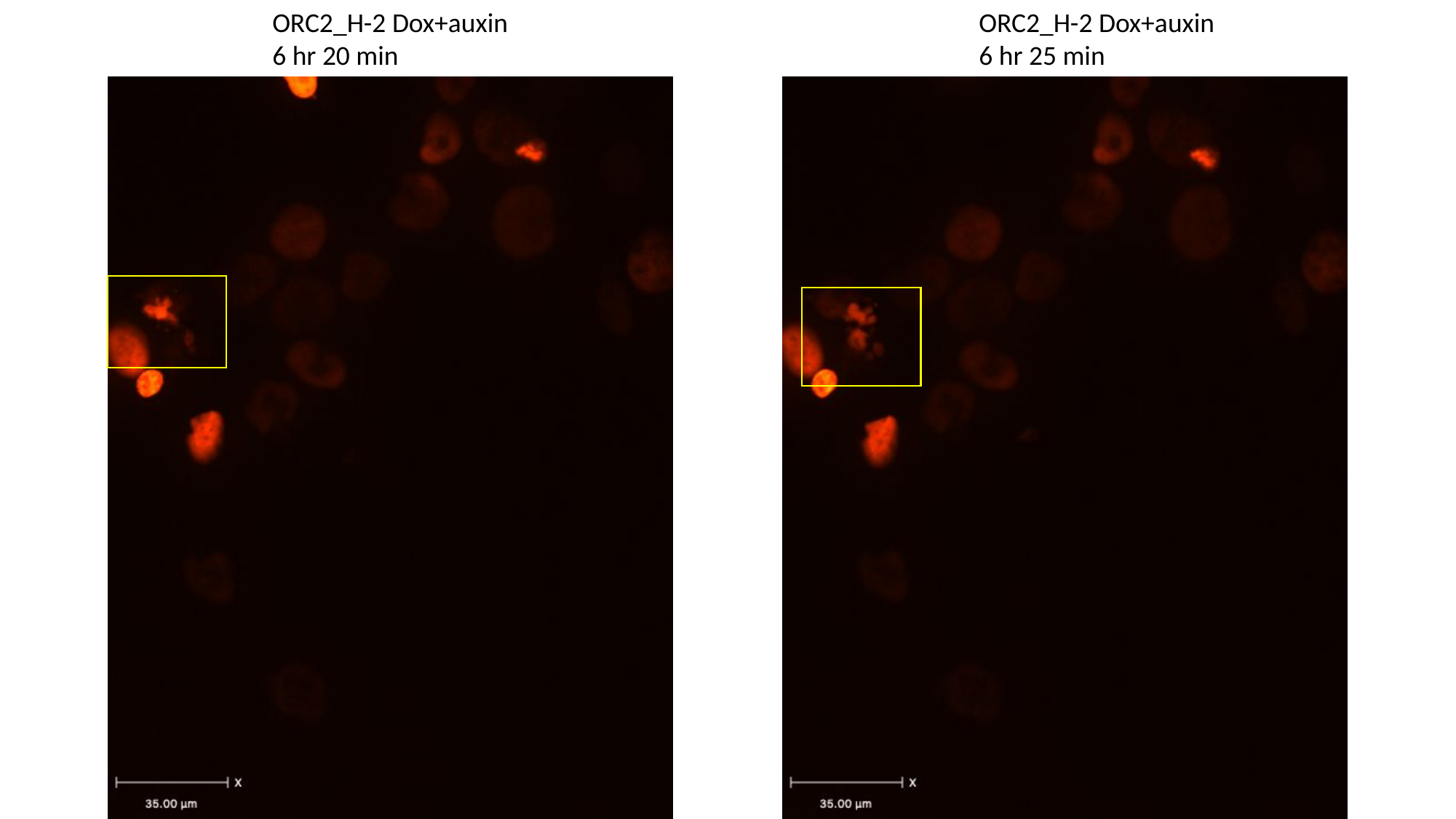

ORC2_H-2 Dox+auxin
6 hr 20 min
ORC2_H-2 Dox+auxin
6 hr 25 min
