# Supplementary material for: The human origin recognition complex is essential for pre-RC assembly, mitosis, and maintenance of nuclear structure
Source: eLife. 2021 Feb 1;10:e61797. doi: 10.7554/eLife.61797 (PMC7877914; doi:10.7554/eLife.61797)
Supplement: Figure 8—source data 1. [file elife-61797-fig8-data1.docx]

|  | HCT116 p53+/+ (n = 422) | | | HCT116 p53-/-  (n = 415) | | | ORC1-/- (B14) (n = 438) | | | ORC2-/- (P44) (n = 437) | | |
| --- | --- | --- | --- | --- | --- | --- | --- | --- | --- | --- | --- | --- |
| Normal | 137 | 138 | 138 | 134 | 136 | 132 | 69 | 76 | 62 | 136 | 137 | 132 |
| Abnormal/ Involuted | 3 | 3 | 2 | 4 | 6 | 2 | 66 | 72 | 59 | 10 | 14 | 7 |
| Nuclear  Giants  w/ or w/o Lamin B1 | 0 | 1 | 0 | 0 | 1 | 0 | 11 | 15 | 8 | 0 | 1 | 0 |

Figure 8 – source data 1

**Figure 8i - Quantitation of Nuclear Phenotypes**

**Statistical analysis: 2way ANOVA with Multiple comparisons (all compared to HCT116 p53-/-)**

|  |  |  |  |  |  |  |  |  |
| --- | --- | --- | --- | --- | --- | --- | --- | --- |
| Within each row, compare columns (simple effects within rows) |  |  |  |  |  |  |  |  |
|  |  |  |  |  |  |  |  |  |
| Number of families | 3 |  |  |  |  |  |  |  |
| Number of comparisons per family | 3 |  |  |  |  |  |  |  |
| Alpha | 0.05 |  |  |  |  |  |  |  |
|  |  |  |  |  |  |  |  |  |
| Šídák's multiple comparisons test | Mean Diff. | 95.00% CI of diff. | Below threshold? | Summary | Adjusted P Value |  |  |  |
|  |  |  |  |  |  |  |  |  |
| Normal |  |  |  |  |  |  |  |  |
| HCT116 p53-/- vs. HCT116 p53+/+ | -3.667 | -10.63 to 3.300 | No | ns | 0.4674 |  |  |  |
| HCT116 p53-/- vs. ORC1-/- (B14 | 65.00 | 58.03 to 71.97 | Yes | **** | <0.0001 |  |  |  |
| HCT116 p53-/- vs. ORC2-/- (P44) | -1.000 | -7.967 to 5.967 | No | ns | 0.9771 |  |  |  |
|  |  |  |  |  |  |  |  |  |
| Abnormal/ Involuted |  |  |  |  |  |  |  |  |
| HCT116 p53-/- vs. HCT116 p53+/+ | 1.333 | -5.633 to 8.300 | No | ns | 0.9484 |  |  |  |
| HCT116 p53-/- vs. ORC1-/- (B14) | -61.67 | -68.63 to -54.70 | Yes | **** | <0.0001 |  |  |  |
| HCT116 p53-/- vs. ORC2-/- (P44) | -6.333 | -13.30 to 0.6332 | No | ns | 0.0827 |  |  |  |
|  |  |  |  |  |  |  |  |  |
| Nuclear Giants w/ or w/o Lamin B1 |  |  |  |  |  |  |  |  |
| HCT116 p53-/- vs. HCT116 p53+/+ | 0.000 | -6.967 to 6.967 | No | ns | >0.9999 |  |  |  |
| HCT116 p53-/- vs. ORC1-/- (B14) | -11.00 | -17.97 to -4.033 | Yes | ** | 0.0014 |  |  |  |
| HCT116 p53-/- (n = 415) vs. ORC2-/- | 0.000 | -6.967 to 6.967 | No | ns | >0.9999 |  |  |  |
|  |  |  |  |  |  |  |  |  |
|  |  |  |  |  |  |  |  |  |
| Test details | Mean 1 | Mean 2 | Mean Diff. | SE of diff. | N1 | N2 | t | DF |
|  |  |  |  |  |  |  |  |  |
| Normal |  |  |  |  |  |  |  |  |
| HCT116 p53-/- vs. HCT116 p53+/+ | 134.0 | 137.7 | -3.667 | 2.715 | 3 | 3 | 1.351 | 24.00 |
| HCT116 p53-/- vs. ORC1-/- (B14) | 134.0 | 69.00 | 65.00 | 2.715 | 3 | 3 | 23.94 | 24.00 |
| HCT116 p53-/- vs. ORC2-/- (P44) | 134.0 | 135.0 | -1.000 | 2.715 | 3 | 3 | 0.3683 | 24.00 |
|  |  |  |  |  |  |  |  |  |
| Abnormal/ Involuted |  |  |  |  |  |  |  |  |
| HCT116 p53-/- vs. HCT116 p53+/+ | 4.000 | 2.667 | 1.333 | 2.715 | 3 | 3 | 0.4911 | 24.00 |
| HCT116 p53-/- vs. ORC1-/- (B14) | 4.000 | 65.67 | -61.67 | 2.715 | 3 | 3 | 22.71 | 24.00 |
| HCT116 p53-/- vs. ORC2-/- (P44) | 4.000 | 10.33 | -6.333 | 2.715 | 3 | 3 | 2.333 | 24.00 |
|  |  |  |  |  |  |  |  |  |
| Nuclear Giants w/ or w/o Lamin B1 |  |  |  |  |  |  |  |  |
| HCT116 p53-/- vs. HCT116 p53+/+ | 0.3333 | 0.3333 | 0.000 | 2.715 | 3 | 3 | 0.000 | 24.00 |
| HCT116 p53-/- vs. ORC1-/- (B14) | 0.3333 | 11.33 | -11.00 | 2.715 | 3 | 3 | 4.052 | 24.00 |
| HCT116 p53-/- vs. ORC2-/- (P44) | 0.3333 | 0.3333 | 0.000 | 2.715 | 3 | 3 | 0.000 | 24.00 |
