# Supplementary material for: The human origin recognition complex is essential for pre-RC assembly, mitosis, and maintenance of nuclear structure
Source: eLife. 2021 Feb 1;10:e61797. doi: 10.7554/eLife.61797 (PMC7877914; doi:10.7554/eLife.61797)
Supplement: Transparent reporting form [file elife-61797-transrepform.docx]

***eLife’s* transparent reporting form**

We encourage authors to provide detailed information *within their submission* to facilitate the interpretation and replication of experiments. Authors can upload supporting documentation to indicate the use of appropriate reporting guidelines for health-related research (see [EQUATOR Network](http://www.equator-network.org/%20)), life science research (see the [BioSharing Information Resource](https://biosharing.org/)), or the [ARRIVE guidelines](http://www.plosbiology.org/article/info:doi/10.1371/journal.pbio.1000412) for reporting work involving animal research. Where applicable, authors should refer to any relevant reporting standards documents in this form.

If you have any questions, please consult our Journal Policies and/or contact us: [editorial@elifesciences.org](mailto:editorial@elifesciences.org).

**Sample-size estimation**

- You should state whether an appropriate sample size was computed when the study was being designed
- You should state the statistical method of sample size computation and any required assumptions
- If no explicit power analysis was used, you should describe how you decided what sample (replicate) size (number) to use

Please outline where this information can be found within the submission (e.g., sections or figure legends), or explain why this information doesn’t apply to your submission:

1. For tiling CRISPR screens, the sample size-estimation can be found in Materials and Methods Pooled sgRNA screening first section.
2. For quantitation of immunofluorescent staining, the sample size-estimation for pATM foci can be found in Figure 3—Figure supplement 3d figure legend. The sample size-estimation for pCHK1 foci can be found in Figure 3—Figure supplement 3e figure legend. The sample size-estimation for CENP-C foci can be found in Figure 4—Figure supplement 1 figure legend.
3. In nuclear volume measurement, the sample-size estimation can be found in Figure 4b figure legend.
4. In Palbociclib synchronization experiment, the sample-size estimation of nuclear volume measurement can be found in Figure 4—Figure supplement 3b figure legend.
5. The sample size estimation for the duration of mitosis in cells can be found in “ORC2 depletion in cells leads to aberrant mitosis” section the main text.
6. For each flow cytometry experiment, the sample-size estimation can be found in Figure legend and Materials and Methods section.
7. For quantitation of nuclear phenotypes, the sample-size estimation can be found in Figure 8i.

**Replicates**

- You should report how often each experiment was performed
- You should include a definition of biological versus technical replication
- The data obtained should be provided and sufficient information should be provided to indicate the number of independent biological and/or technical replicates
- If you encountered any outliers, you should describe how these were handled
- Criteria for exclusion/inclusion of data should be clearly stated
- High-throughput sequence data should be uploaded before submission, with a private link for reviewers provided (these are available from both GEO and ArrayExpress)

Please outline where this information can be found within the submission (e.g., sections or figure legends), or explain why this information doesn’t apply to your submission:

1. For tiling CRISPR screens, the number of replicates can be found in First section of results, and the Pooled sgRNA screening section in Materials and Methods. Result of all of them can be found in corresponding Figure or Figure supplement.
2. For all GFP competition assays, the number of replicates can be found in GFP competition and sgRNA complementation assay section in Materials and Methods**.**
3. For cell proliferation rate experiment, the number of replicates can be found in Figure 3 figure legend.
4. For cell cycle analysis experiment, the number of replicates can be found in Cell cycle analysis and pulse EdU label section in Materials and Methods.

**Statistical reporting**

- Statistical analysis methods should be described and justified
- Raw data should be presented in figures whenever informative to do so (typically when N per group is less than 10)
- For each experiment, you should identify the statistical tests used, exact values of N, definitions of center, methods of multiple test correction, and dispersion and precision measures (e.g., mean, median, SD, SEM, confidence intervals; and, for the major substantive results, a measure of effect size (e.g., Pearson's r, Cohen's d)
- Report exact p-values wherever possible alongside the summary statistics and 95% confidence intervals. These should be reported for all key questions and not only when the p-value is less than 0.05.

Please outline where this information can be found within the submission (e.g., sections or figure legends), or explain why this information doesn’t apply to your submission:

1. For tiling CRISPR screens, the statistical information can be found in Quantification and analysis of screen data section in Materials and Methods.
2. For GFP competition assays, the statistical information in each experiment can be found in Figure 2 and Figure 8 figure legends.
3. For nuclear volume measurement, the statistical information can be found in Figure 4b figure legend.
4. For quantitation of nuclear phenotypes, the statistical information can be found in Figure 8i figure legend.

(For large datasets, or papers with a very large number of statistical tests, you may upload a single table file with tests, Ns, etc., with reference to sections in the manuscript.)

**Group allocation**

- Indicate how samples were allocated into experimental groups (in the case of clinical studies, please specify allocation to treatment method); if randomization was used, please also state if restricted randomization was applied
- Indicate if masking was used during group allocation, data collection and/or data analysis

Please outline where this information can be found within the submission (e.g., sections or figure legends), or explain why this information doesn’t apply to your submission:

Not applicable

**Additional data files (“source data”)**

- We encourage you to upload relevant additional data files, such as numerical data that are represented as a graph in a figure, or as a summary table
- Where provided, these should be in the most useful format, and they can be uploaded as “Source data” files linked to a main figure or table
- Include model definition files including the full list of parameters used
- Include code used for data analysis (e.g., R, MatLab)
- Avoid stating that data files are “available upon request”

Please indicate the figures or tables for which source data files have been provided:

Figure 1 – source data 1. Numerical data table for ORC1 tiling sgRNA CRISPR screen log fold depletion in Figure 1a.

Figure 1 – source data 2. Numerical data table for ORC1 tiling sgRNA CRISPR screen log fold depletion in Figure 1b.

Figure 2 – source data 1. Numerical data table for ORC2 tiling sgRNA CRISPR screen log fold depletion in Figure 2a.

Figure 2 – source data 2. Numerical data table for ORC1 tiling sgRNA CRISPR screen log fold depletion in Figure 2b.

Figure 3 – source data 1. Entire films of the cropped western blots in Figure 3a.

Figure 3 – source data 2. Entire films of the cropped western blots in Figure 3g.

Figure 3 – source data 3. Uncropped immunofluorescence image of Figure 3h.

Figure 3 – source data 4. Uncropped immunofluorescence image of Figure 3i.

Figure 3 – source data 5. Entire films of the cropped western blots in Figure 3 – Figure supplement 3a.

Figure 3 – source data 6. Uncropped immunofluorescence image of Figure 3 – Figure supplement 3f.

Figure 4 – source data 1. Uncropped immunofluorescence image of Figure 4a.

Figure 4 – source data 2. Numerical data table for nuclear volume of Figure 4b.

Figure 4 – source data 3. Uncropped immunofluorescence image of Figure 4c.

Figure 7 – source data 1. Numerical data table for p-H3S10 flow cytometry in Figure 7a.

Figure 7 – source data 2. Uncropped immunofluorescence image of Figure 7b.

Figure 7 – source data 3. Uncropped immunofluorescence image of Figure 7c.

Figure 7 – source data 4. Uncropped immunofluorescence image of Figure 7d.

Figure 7 – source data 5. Uncropped immunofluorescence image of Figure 7e.

Figure 8 – source data 1. Numerical data table and statistical analysis for graph in Figure 8i.

Supplement file 1. The sequences of all guide RNAs used for gene editing, including those directed to ORC1-6 and CDC6 as well as positive and negative guides for the tiling CRISPR screens.

Supplement file 2. Sequence of Barcode primers used for Next Gene Sequencing analysis in tiling CRISPR screens.

Supplement file 3. Primers used for exon analysis qPCR of the *ORC2* gene cDNAs from various cell lines.
